# Overexpression of the double homeodomain protein DUX4c interferes with myofibrillogenesis and induces clustering of myonuclei

**DOI:** 10.1186/s13395-017-0148-4

**Published:** 2018-01-12

**Authors:** Céline Vanderplanck, Alexandra Tassin, Eugénie Ansseau, Sébastien Charron, Armelle Wauters, Céline Lancelot, Kelly Vancutsem, Dalila Laoudj-Chenivesse, Alexandra Belayew, Frédérique Coppée

**Affiliations:** 1 Laboratory of Molecular Biology, Research Institute for Health Sciences and Technology, University of Mons, 6, Avenue du Champs de Mars, B-7000 Mons, Belgium; 2 INSERM U1046, CHU A. de Villeneuve, University of Montpellier, 34295 Montpellier, France

**Keywords:** Disorganized myotubes, Myonuclear clustering, Cytoskeleton, β-catenin, Proliferation, Differentiation

## Abstract

**Background:**

Facioscapulohumeral muscular dystrophy (FSHD) is associated with DNA hypomethylation at the 4q35 D4Z4 repeat array. Both the causal gene *DUX4* and its homolog *DUX4c* are induced*.* DUX4c is immunodetected in every myonucleus of proliferative cells, while DUX4 is present in only 1/1000 of myonuclei where it initiates a gene deregulation cascade. FSHD primary myoblasts differentiate into either atrophic or disorganized myotubes. DUX4 expression induces atrophic myotubes and associated FSHD markers. Although DUX4 silencing normalizes the FSHD atrophic myotube phenotype, this is not the case for the disorganized phenotype. DUX4c overexpression increases the proliferation rate of human TE671 rhabdomyosarcoma cells and inhibits their differentiation, suggesting a normal role during muscle differentiation.

**Methods:**

By gain- and loss-of-function experiments in primary human muscle cells, we studied the DUX4c impact on proliferation, differentiation, myotube morphology, and FSHD markers.

**Results:**

In primary myoblasts, DUX4c overexpression increased the staining intensity of KI67 (a proliferation marker) in adjacent cells and delayed differentiation. In differentiating cells, DUX4c overexpression led to the expression of some FSHD markers including β-catenin and to the formation of disorganized myotubes presenting large clusters of nuclei and cytoskeletal defects. These were more severe when DUX4c was expressed before the cytoskeleton reorganized and myofibrils assembled. In addition, endogenous DUX4c was detected at a higher level in FSHD myotubes presenting abnormal clusters of nuclei and cytoskeletal disorganization. We found that the disorganized FSHD myotube phenotype could be rescued by silencing of DUX4c, not DUX4.

**Conclusion:**

Excess DUX4c could disturb cytoskeletal organization and nuclear distribution in FSHD myotubes. We suggest that DUX4c up-regulation could contribute to DUX4 toxicity in the muscle fibers by favoring the clustering of myonuclei and therefore facilitating DUX4 diffusion among them. Defining DUX4c functions in the healthy skeletal muscle should help to design new targeted FSHD therapy by DUX4 or DUX4c inhibition without suppressing DUX4c normal function.

**Electronic supplementary material:**

The online version of this article (10.1186/s13395-017-0148-4) contains supplementary material, which is available to authorized users.

## Background

Facioscapulohumeral muscular dystrophy (FSHD) is one of the most common neuromuscular disorders [1]. It is characterized by muscle weakness and atrophy of the facial muscles, progressing to the upper arms, shoulder girdle, and lower limbs. FSHD is linked to the D4Z4 repeat array on 4q35 and requires both genetic and epigenetic conditions allowing the expression of *DUX4* (double homeobox 4), a gene our group has identified in each D4Z4 repeat unit [2–4] (reviewed by [5]). The genetic condition is characterized by a SNP producing a polyadenylation signal in the pLAM region 3′ of the distal D4Z4 unit, leading to the expression of a stable mRNA and toxic DUX4 protein [4, 6]. The epigenetic condition is D4Z4 DNA hypomethylation, allowing *DUX4* gene expression (reviewed by [7, 8]). This results either from contraction of the D4Z4 repeat array, as observed in the major FSHD1 form (OMIM#158900), or loss-of-function mutations of *SMCHD1* or *DNMT3B*, involved in the much rarer FSHD2 form (OMIM#614982), which is clinically undistinguishable from FSHD1 (reviewed by [9]). The level of D4Z4 DNA hypomethylation generally correlates with disease variability and severity [10], as observed in severely affected individuals with inherited combined FSHD1 and 2 conditions [11, 12]. DUX4 encodes a 52-kDa transcription factor that is normally expressed in germline cells and is activated in a small proportion of FSHD muscle nuclei, from which it diffuses and initiates a transcription deregulation cascade leading to muscle atrophy, muscle differentiation defects, and oxidative stress, which are key features of FSHD [3, 4, 13–21]. The occurrence of modulators of DUX4 toxicity is suggested by the observation of two phenotypes in FSHD primary cell cultures, where myotubes are either very thin and atrophic (aFSHD) or large and disorganized with clusters of nuclei (dFSHD). The two phenotypes are found in different proportions in different myoblast primary “lines” [16, 22]. In healthy primary myotubes, DUX4 is expressed at a very low level in < 1% myonuclei, in contrast to its expression in approximately 8% (dFSHD) to 10% (aFSHD) of myonuclei in FSHD cultures. Moreover, DUX4 is induced approximately threefold in aFSHD compared to the healthy muscle cells [16, 23]. Surprisingly, DUX4 inhibition prevents the formation of atrophic but not disorganized FSHD myotubes [24]. When embryonic stem cells (hESC) with the FSHD1 genetic defect were differentiated to skeletal muscle cells, those which expressed DUX4 exhibited defects reported in FSHD cells and formed thinner myotubes, similar to aFSHD ones [25].

We have also characterized a gene, mapped 42-kb centromeric of the proximal D4Z4 element, in a truncated and inverted D4Z4 unit that encodes a DUX4 homologous protein, which we have designated DUX4c [26, 27]. DUX4c is a transcription factor that is identical to DUX4, except that it is 50 residues shorter, and its last 32 residues share 40% identity with DUX4. DUX4c is detected in primary healthy myoblasts and is induced upon their differentiation and is up-regulated together with DUX4 in FSHD muscle cells and biopsies [27, 28]. The muscles of patients with Duchenne muscular dystrophy (DMD) exhibit a high regeneration rate and increased DUX4c levels, with strong DUX4c immunolabeling being observed in some desmin-positive regenerating fibers [27, 28]. DUX4c overexpression increases the proliferation rate of human TE671 myoblasts and inhibits the differentiation of both human TE671 and mouse C2C12 myoblasts [27, 29]. This inhibition is rescued by overexpression of the myogenic factors MyoD and Myf5 in C2C12 myoblasts [29]. DUX4c induces (and interacts with) the MYF5 protein in mouse and human myoblasts [27]. DUX4c overexpression also induces myogenic microRNAs, and DUX4c knockdown causes stronger inhibition of these miRNAs in FSHD compared with healthy myotubes [30]. All of these data support a role for DUX4c during the proliferation and differentiation of healthy muscle cells and suggest that its abnormal induction could contribute to FSHD muscle pathology. A recent transcriptomic study supports the role of DUX4c in muscle development and disease [31].

In the present study, we investigated the role of DUX4c in human myotube phenotypes. Its overexpression in myotubes caused differentiation defects characterized by an abnormal troponin T distribution and large clusters of nuclei, leading to a disorganized phenotype. In contrast, DUX4c inhibition in FSHD myoblasts prevented the formation of disorganized myotubes. These gain- and loss-of-function experiments support a possible contribution of DUX4c to the FSHD pathophysiology.

## Methods

### Myogenic cell culture, transfections, and induction

Primary human myoblasts from an unaffected control and a patient with FSHD were isolated from muscle biopsies, purified, and established as described previously [22]. The myoblasts were grown in 35-mm collagen-coated dishes (Ywaki, Japan) in DMEM with 4.5 g/l glucose and L-glutamine (Lonza) as well as gentamicin (50 μg/ml, Sigma-Aldrich), 10% fetal bovine serum (FBS) (Invitrogen), and 1% Ultroser G (Pall BioSepra, Cergy-St-Christophe, France) at 37 °C under 5% CO_2_. Confluent myoblast cultures were differentiated by switching the medium to DMEM/gentamicin (50 μg/ml) with 2% FBS. Myoblasts were transfected in their culture medium 24 h after seeding, with Fugene HD (Roche Diagnostics) and DNA at a ratio of 6:2 (μl of Fugene HD:μg of DNA), according to the manufacturer’s instructions [32].

The vectors were *pCIneo* (Promega), *pCIneo-DUX4c*, *pAC1M2-DUX4c*, *p3-kb-DUX4c*, and *p7.5-kb-DUX4c* [27]. Doxycycline induction was performed as previously described [27].

### Transfection with siRNA

Three siRNAs were designed against the *DUX4c 3′UTR* sequence (custom siRNA design, Ambion). Among these, siRNA1 (5′-ccagagtttcagcaaaagg-3′) was previously described for its efficiency to inhibit DUX4c overexpression and its specificity as it did not interfere with DUX4 expression [27]. For cell transfection, we used the “Silencer siRNA Starter Kit” (Ambion) with the “*SiPORT NeoFX*” transfection agent, as described in [32]. We used 4 μl of *siPORTNeoFX* and 10 nM siRNA for primary human myoblasts. All transfections were performed in duplicate wells and were repeated three times to ensure consistency.

### RT-qPCR and 3′RACE

Primary myoblasts were harvested during proliferation or 1 day after the induction of differentiation. Total RNA was extracted and retro-transcribed, and quantitative PCR was performed using a specific γ-Catenin TaqMan assay (Hs00158408_m1, ThermoFisher Scientific). The quantity of γ-Catenin mRNA is provided relative to its expression level in healthy cells, which was set to 1. The means and standard errors are indicated. The 3′RACE experiments were performed as previously described [27]. The PCR products were cloned and sequenced.

### Immunofluorescence

Human primary myoblasts were fixed in PBS containing 4% paraformaldehyde (Sigma-Aldrich) and treated with PBS/0.5% Triton X-100. After blocking in PBS/20% FBS, the cells were incubated with primary antibodies for 2 h at room temperature. The following antibodies and dilutions were used: mouse monoclonal (mAb) anti-troponin T 1/100 (clone JLT-12, Sigma-Aldrich), rabbit polyclonal anti-β-catenin 1/200 (ECM Biosciences), anti-KI67 1/200 (mAb, Abcam), anti-α-Tubulin mAb 1/500 (Abcam), rabbit polyclonal anti-αB-crystallin 1/50 (Enzo Life Sciences), mAb 9A12 (which we developed against DUX4) 1/50 ([4]; clone 9A12, Merck Millipore), and rabbit antiserum directed against a DUX4c peptide 1/150 [27]. After washing and blocking, cells were incubated for 1 h at room temperature with Alexa Fluor secondary antibodies at 1/100 (goat anti-mouse 488 and anti-rabbit 555, Invitrogen) then washed again and mounted with Vectashield mounting medium containing DAPI (Vector Laboratories, Burlingame, Biosciences).

Images were acquired with either a Nikon Eclipse i20 microscope equipped with filters allowing the detection of proteins showing low expression and with a DS-U3 DS camera control unit at room temperature. Plan Fluor 20 X, Plan Fluor 409, and 609 Apo-VC high-resolution oil immersion objectives were used, with 350, 480, and 540 nm excitation for the 4,6-diamidino-2-phenylindole (DAPI), fluorescein isothiocyanate (FITC), and tetramethylrhodamine isothiocyanate (TRITC) channels, respectively. The Nikon Instrument Software NIS-elements Basic Research analysis software was used both for image acquisition and for measure of KI67 staining intensity per nucleus. ImageJ was used for image merging.

### Immunodetection through Western blotting

Cells were lysed in hypertonic buffer containing 50 mM Tris, pH 7, 50 mM NaCl, 0.1% NP40, a protease inhibitor cocktail (Roche Diagnostics), and 1 mM DTT. Each cell lysate was separated via electrophoresis in a 10 or 12% polyacrylamide gel in the presence of SDS, followed by transfer to a nitrocellulose membrane (GE Healthcare Europe GmbH, Diegem, Belgium). Protein transfer was confirmed through Ponceau red staining. The blot was blocked with 5% non-fat dry milk diluted in PBS for 1 h at room temperature. The membranes were then incubated at 4 °C overnight with primary antibodies in PBS/2% BSA. The following antibodies and dilutions were used: mAb 9A12 1/1000, rabbit DUX4c antiserum 1/1000, anti-troponin T mAb 1/1000 (clone JLT-12, Sigma-Aldrich), rabbit polyclonal anti-β-catenin 1/1000 (ECM Biosciences, KY, USA), anti-JUP 1/1000 (ECM Biosciences, KY, USA), rabbit polyclonal anti-MYF5 1/500 (Santa Cruz Biotechnologies), rabbit polyclonal anti-Atrogin-1 1/1000 (or anti-Atrogin-1/MAFbx, ECM Biosciences), rabbit polyclonal anti-MuRF1 1/1000 (ECM Biosciences), anti-CRYM mAb 1/1000 (or anti-mu-crystallin, Abnova, Heidelberg, Germany), and anti-TP53 mAb 1/1000 (Abcam, Cambridge, UK). The membranes were subsequently washed in PBS and incubated with secondary antibodies coupled to horseradish peroxidase (HRP) 1/10000 (GE Healthcare) for 1 h at room temperature. Proteins were detected on Amersham Hyperfilm ECL (GE Healthcare) with the LiteAbLot (Euroclone, Victoria, Australia), Lumilight (Roche Diagnostics), or Super signal West Femto maximum sensitivity substrate kit (Thermo Scientific). For standardization, the membranes were stripped (200 nM glycine, 0.5 M NaCl, 0.1% SDS, pH 2.2), and immunostaining was performed with rabbit polyclonal anti-actin serum 1/1000 (Sigma-Aldrich), followed by HRP-coupled secondary antibodies 1/10,000 (GE Healthcare). Densitometry of the immunoreactive bands was performed with LabImage 1D Software (Kapelan Bio-Imaging) or the Thermo Scientific MyImageAnalysis Software v1.1. The data are normalized to control loading levels in each sample.

### Statistical analyses

Statistical significance was evaluated using Student’s *t* test, except for KI67 immunofluorescence in gain-of-function experiment for which the Mann-Whitney test was used.

## Results

### DUX4c overexpression in myoblasts induces the disorganized myotube phenotype

DUX4c overexpression inhibits the differentiation of C2C12 and TE671 myoblasts into myotubes [27, 29]. We transfected primary human myoblasts with a DUX4c expression vector (*pCIneo-DUX4c*) and switched the cells to differentiation medium. Three days later, the cell morphology differed between the healthy myocytes (transfected with the empty *pCIneo* vector), which were elongated and aligned to fuse, and the DUX4c-overexpressing cells, which remained rounder and had not aligned (data not shown), as we previously observed when DUX4c was overexpressed in TE671 cells [27]. After 6 days of differentiation, healthy myotubes exhibited normally aligned nuclei, whereas DUX4c-positive myonuclei formed clusters in enlarged and deformed myotubes (Additional file 1: Figure S1A). A similar phenotype was observed in FSHD myotubes, showing cytoplasmic staining of endogenous DUX4c with patches of troponin T accumulation (Additional file 1: Figure S1B). We previously observed similar endogenous DUX4c staining in dFSHD myotubes and FSHD muscle sections in association with the clustering of myonuclei [24, 28].

### DUX4c overexpression induces troponin T delocalization

**Fig. 1:**
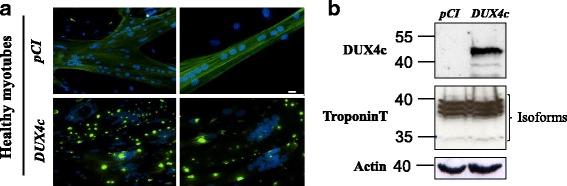
DUX4c overexpression induces misdistribution of troponin T and abnormal distribution of myonuclei. Healthy human primary myotubes were transfected with *pCIneo-DUX4c* or the empty *pCIneo* expression vector and switched to differentiation medium. **a** Five days later, troponin T was detected by immunofluorescence (green), and nuclei were labeled with DAPI (blue). Scale bar: 20 μM. **b** A 20 μg sample of total protein extracted from the same cells was separated via electrophoresis (PAGE-SDS), then transferred to a Western blot, immunodetected with the indicated primary antibodies and appropriate secondary antibodies coupled to HRP, and revealed through chemiluminescence

We transfected primary human myoblasts as above but switched the culture to differentiation medium at an earlier time point (5 h instead of 24 h). Five days later, DUX4c-overexpressing myotubes exhibited clusters of nuclei and local dense troponin T staining, instead of striations across the entire cytoplasm as observed in healthy myotubes (Fig. 1a). However, global troponin T protein levels were not affected by DUX4c overexpression, as shown by immunodetection with a specific antibody in Western blots prepared with total cell extracts (Fig. 1b).

### DUX4c overexpression in myotubes induces a disorganized phenotype

**Fig. 2:**
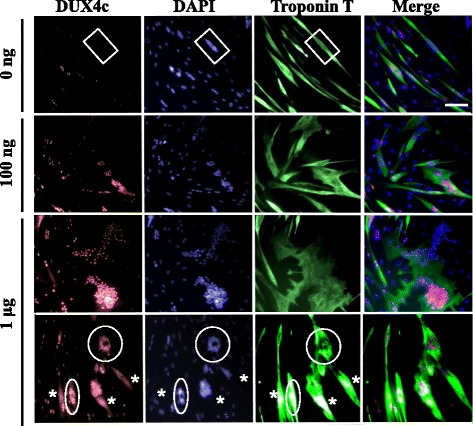
DUX4c overexpression induces the formation of disorganized myotubes with large nuclear clusters. Healthy primary myoblasts were transfected with *pAC1M2-DUX4c* and differentiated 48 h later. After the first myotubes formed (day 3), DUX4c expression was induced by the addition of 100 ng or 1 μg of doxycycline to the culture medium. At day 8, myotubes were fixed, and DUX4c (red) and troponin T (green) were immunodetected. Nuclei were labeled with DAPI. A cluster of nuclei (> 5 nuclei) without endogenous DUX4c labeling is boxed (0 ng Dox). Clusters of nuclei presenting a ring-like structure are circled. Stars indicate cytoplasmic DUX4c partially co-localizing with troponin T. Scale bar: 30 μm

In the previous experiments, we expressed DUX4c in myoblasts before or at the beginning of differentiation. To evaluate the impact of DUX4c expression on already-formed myotubes, we transfected primary myoblasts with the doxycycline-inducible *pACIM2-DUX4c* expression vector [27] and switched the cells to differentiation medium. Three days later, we induced DUX4c expression with different doses of doxycycline, fixed the cells after an additional 5 days, and immunodetected DUX4c and troponin T. Without doxycycline, basal nuclear DUX4c staining was observed in cells with clusters of two to five nuclei, suggesting recent fusion or myoblasts about to fuse (Fig. 2, top panels), as previously observed [28]. With increasing amounts of doxycycline, the myotubes appeared progressively larger and more deformed. They contained clusters of DAPI-stained nuclei more frequently and included more nuclei with increasing DUX4c expression. Cytoplasmic DUX4c partially co-localizing with troponin T was also detected on one side of the myotubes (Fig. 2 bottom panels and Additional file 2: Figure S2, asterisks) or at one of their tips (Additional file 2: Figure S2 circle), as previously observed [28]. In some myotubes, clusters of DUX4c-positive nuclei formed rings; cytoplasmic troponin T immunofluorescence was observed within these structures and, more diffusely, around them (Fig. 2, Additional file 2: Figure S2). We did not observe intense spots of troponin T as in Fig. 1, but its organization was disturbed next to the large or ring-like clusters of nuclei (Fig. 2, Additional file 2: Figure S2).

### β-catenin accumulates in disorganized FSHD myotubes and upon DUX4c overexpression

As abnormal β-catenin accumulation causes oversized and disorganized myotubes with clusters of nuclei [33], we examined the amount of β-catenin in a Western blot prepared with total extracts from myotubes in which DUX4c had been induced (a parallel culture is shown in Fig. 2). An increase in β-catenin was immunodetected (2.3-fold under the highest dose of doxycycline) (Additional file 3: Figure S3).

**Fig. 3:**
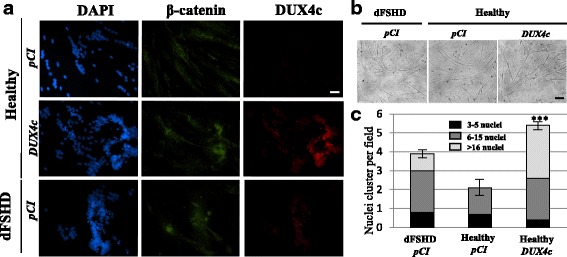
DUX4c overexpression induces abnormal clusters of myonuclei and misdistribution of β-catenin. **a** FSHD and healthy primary myoblasts were transfected with the empty *pCIneo* or *pCIneo-DUX4c* expression vector, and differentiation was induced 24 h later. Immunofluorescence detection with antibodies against β-catenin (green) or DUX4c (red) was performed at day 3. Nuclei were labeled with DAPI. Scale bar: 20 μm. **b** Representative field from each culture (white light). Scale bar: 50 μm. **c** The nuclei per cluster were counted in each culture (10 fields per culture) and are presented as the mean ± SD. The proportion of nuclei present per cluster is shown in grayscale. Nuclear clusters with more than 16 nuclei were seldom found in healthy myotubes; the mean is therefore close to zero. ***p < 0.001

**Fig. 4:**
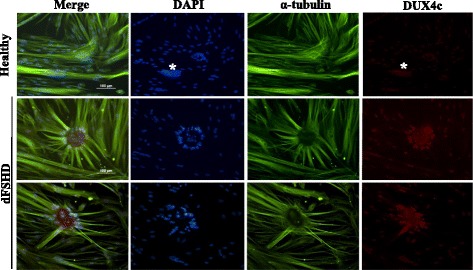
dFSHD myotubes present abnormal clusters of nuclei overexpressing DUX4c as well as cytoplasmic DUX4c and α-tubulin disorganization. Healthy and FSHD primary myoblasts were differentiated, then fixed 6 days later, and DUX4c (red) and α-tubulin (green) were detected by immunofluorescence. A large cluster of myonuclei (> 16, indicated by *) can be observed, while such clusters were very seldom found in healthy cultures. Scale bars: 100 μm

We then examined the intracellular location of β-catenin through immunofluorescence. In healthy primary human myotubes, the diffuse β-catenin staining observed excluded the nuclei (Fig. 3a top panels). In healthy myotubes overexpressing DUX4c and in dFSHD myotubes, β-catenin especially accumulated in the vicinity of nuclear clusters (Fig. 3a middle and bottom panels), and myotube morphology was similar in the two types of cultures (Fig. 3b). An average of four and five clusters of nuclei were counted per field in dFSHD and in DUX4c-overexpressing myotubes, respectively, while only two clusters were detected in healthy myotubes. In addition, approximately 50 and 25% of these clusters contained more than 16 nuclei in DUX4c-overexpressing and dFSHD myotubes, respectively. Healthy myotubes seldom presented such large clusters (Figs. 3c and 4).

### High levels of nuclear and cytoplasmic DUX4c are associated with clusters of nuclei in disorganized FSHD myotubes

After 6 days in differentiation medium, nuclei were observed in ring-shaped clusters in dFSHD myotubes, but not in a parallel healthy culture (Fig. 4). Similar ring-forming nuclei were detected in DUX4c-overexpressing myotubes after 8 days of differentiation (Fig. 2, Additional file 2: Figure S2). In dFSHD myotubes, such structures were associated with strong DUX4c immunofluorescence in nuclei surrounding a large area of cytoplasmic DUX4c staining. Several DAPI-labeled spots that were apparently smaller than nuclei were present within these nuclear rings. Intriguingly, filaments of α-tubulin seemed to converge toward these nuclear ring clusters, forming flower-like structures. As shown above, in DUX4c-overexpressing myotubes with delocalized troponin T (Figs. 1 and 2, Additional file 2: Figure S2), α-tubulin appeared disorganized (Additional file 4: Figure S4A top) or almost absent (Additional file 4: Figure S4A bottom, boxed) in the vicinity of nuclear clusters presenting strong DUX4c immunofluorescence. The nuclei in these clusters were generally smaller than the other nuclei in the same culture (Fig. 4, Additional file 4: Figure S4A). The dFSHD myotubes with delocalized α-tubulin also presented α-crystallin B chain immunofluorescence in the cytoplasm at a much higher intensity than healthy myotubes (Additional file 4: Figure S4B).

### DUX4c expression induces FSHD markers

As DUX4c shares a high sequence identity with DUX4 (including the DNA binding homeodomain region), we investigated whether DUX4c could induce the expression of genes we have previously shown to be induced in FSHD muscle cells or through DUX4 expression [32]. We transfected myoblasts with DUX expression vectors and performed immunodetection on Western blots 48 h later. DUX4c overexpression induced CRYM as did DUX4, but not P53 (Additional file 5: Figure S5A). In the myotubes obtained after 8-day differentiation, we then immunodetected the E3 ubiquitin ligases MuRF1 and Atrogin-1 (also known as TRIM63 or F-box only protein 32, respectively) associated with muscle atrophy. *DUX4c* overexpression had not affected the level of Atrogin-1 (in contrast to DUX4) (Additional file 5: Figure S5B) but delocalized MuRF1 in the nucleus as observed following DUX4 overexpression or in FSHD myotubes (Additional file 5: Figure S5C and [32]). This is in contrast to the global MuRF1 weak staining detected in healthy myotubes (Additional file 5: Figure S5C and [32]).

Similar to the above results in myotubes (Fig. 3a), FSHD primary myoblasts presented higher levels of β-catenin, which might be attributed to DUX4 or DUX4c overexpression (Additional file 5: Figure S5B). We also observed a significant increase in mRNA expression of the β-catenin homolog γ-catenin (also known as JUP/Junction Plakoglobin). Indeed primary (aFSHD and dFSHD) as well as immortalized FSHD myoblasts presented higher *JUP* mRNA levels in proliferation and 1 day after the induction of differentiation as compared to healthy cells (Additional file 5: Figure S5D and data not shown). There was no regulation of *JUP* mRNA between proliferation and early differentiation in either cell line. At the protein level, a JUP induction in the FSHD sample or following DUX4 or DUX4c expression was less clear (stars in Additional file 5: Figure S5B), even though the gene encoding JUP has been described as a direct DUX4 target [14], and its mRNA level rapidly increases upon DUX4 induction in C2C12 cells [13].

### DUX4c gain and loss of function affect myoblast proliferation.

**Figure 5:**
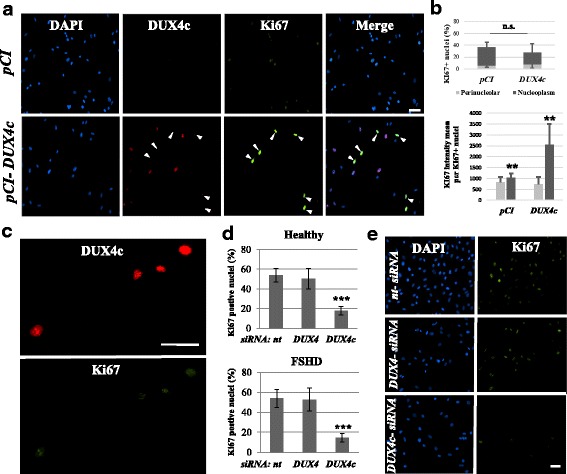
DUX4c gain and loss of function affect cell proliferation. **a** Representative fields of healthy myoblasts transfected with the indicated *pCIneo* expression vectors, fixed 24 h later, and processed for immunofluorescence detection of DUX4c (red) and KI67 (green). Arrows point to KI67-positive cells. **b** The percentage of perinucleolar and nucleoplasmic KI67-positive nuclei on total nuclei was counted in myoblasts showed in **a**. and represented as the mean ± SD. Either 10 or 15 fields were counted for myoblasts transfected either with *pCIneo* or *pCIneo-DUX4c*, respectively (total number of nuclei: 685 and 433, respectively). In addition, the perinucleolar and nucleoplasmic KI67 intensity were measured in each field. The total perinucleolar or nucleoplasmic KI67 intensity (all fields) was divided by the total number of KI67-positive nuclei (either perinucleolar or nucleoplasmic, respectively), and represented as the mean ± SD. The number of nucleoplasmic KI67-positive nuclei was 35 in the healthy culture and 31 in the FSHD culture. For the significance, a Mann-Whitney test was applied. **p < 0.01 (**c**). Magnification of DUX4c-expressing cells with partial co-localization of nucleolar KI67. **d** The number of KI67-positive nuclei was counted in 10 fields of healthy and FSHD myoblasts. The percentage of KI67-positive nuclei is represented as the mean ± SD. ***p < 0.001 was considered significant. **e** Representative fields of FSHD myoblasts transfected with the indicated siRNAs, then fixed 24 h later, and processed for the immunofluorescence detection of KI67. Nuclei were stained with DAPI. Scale bar: 30 μm

We previously showed that DUX4c overexpression induces the proliferation of TE671 muscle cells and inhibits their differentiation [27]. In a similar experiment with healthy primary myoblasts, we detected induction of the KI67 proliferation marker (Fig. 5a). Intriguingly, the cells exhibiting the strongest nucleoplasmic KI67 labeling did not correspond to DUX4c-overexpressing cells (Fig. 5a). The number of KI67-positive nuclei did not differ between muscle cells transfected with a backbone or a DUX4c-expression vector. Nevertheless, the mean intensity of nucleoplasmic KI67 staining per nucleus was 2.5-fold higher following DUX4c overexpression (*p* < 0.01) (Fig. 5b). We also observed that a few DUX4c-overexpressing cells presented KI67 staining, mostly in nuclear spots that could be nucleoli (Fig. 5c).

Three siRNAs targeting the *DUX4c* 3′UTR (region of lowest similarity with *DUX4* sequence) were synthesized based on the *DUX4c* mRNA ends found either in C2C12 cells transfected with a 7.5-kb genomic vector including the *DUX4c* gene and natural promoter [27] or in FSHD primary myoblasts (Additional file 6: Figure S6A, B). However, DUX4c could only be knocked down using siRNA1 (Additional file 6: Figure S6C–E and [27]). We then transfected healthy and FSHD primary myoblasts with siRNAs specific to either *DUX4c* [27] or *DUX4* [32] mRNA, or with non-targeted (*nt*) siRNA (Additional file 7: Figure S7). Twenty-four hours later, we evaluated myoblast proliferation by counting the number of mitotic events in the culture dishes. In the presence of the *DUX4* or *nt* siRNA, both healthy and FSHD myoblasts exhibited five to six mitotic events per field. In contrast, myoblasts treated with the *DUX4c* siRNA exhibited an average of only one mitotic event per field (Additional file 8: Figure S8). In accord with a reduction of cell proliferation, a significant decrease in KI67-positive nuclei (staining observed during the late G1, S, G2, and M phases) was observed in myoblasts treated with the *DUX4c* siRNA compared with the *DUX4* or *nt* siRNA (Fig. 5d, e).

### DUX4c silencing suppresses the FSHD disorganized myotube phenotype

**Fig. 6:**
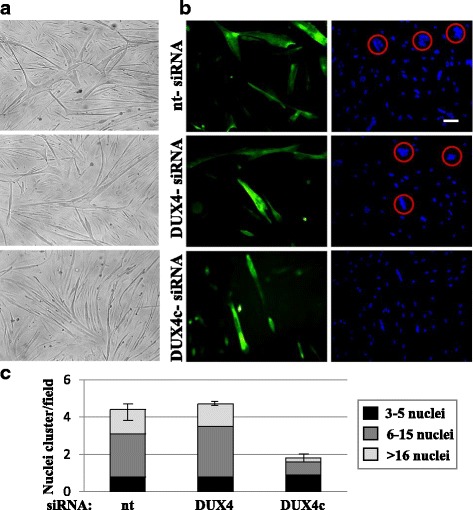
RNA interference against DUX4c suppresses the disorganized myotube phenotype. dFSHD primary myoblasts were transfected with the indicated siRNAs and differentiated. **a** At day 8, microscope pictures were obtained under white light. **b** The cells were then fixed, and troponin T (green) was detected by immunofluorescence. Nuclei were stained with DAPI. Scale bar: 30 μm. **c** The average (and SD) numbers of nuclei per cluster and clusters per field were counted (10 fields per culture). The proportion of nuclei per cluster is shown in grayscale. ***p < 0.001 was considered significant

We transfected dFSHD myoblasts with the *DUX4c*, *DUX4*, or *nt* siRNA and switched the culture to differentiation medium. Eight days later, all of the cultures exhibited myotubes. Large deformed myotubes highlighted by troponin T staining were observed in the presence of the *DUX4* or *nt* siRNA, which showed large clusters of myonuclei. In contrast, treatment with the *DUX4c* siRNA resulted in a return to a “normal” morphology, with thinner myotubes and a greater number of aligned myonuclei (Fig. 6a, b). The numbers of nuclei per cluster and clusters per field were counted in each of the three cultures: an average of four and five clusters per field were visible in the cultures of disorganized FSHD myotubes treated with the *nt* siRNA or the *DUX4* siRNA, respectively, while an average of two clusters were counted in myotubes treated with the *DUX4c* siRNA (Fig. 6c).

## Discussion

Overexpression of DUX4, the FSHD causal gene, was previously linked to muscle atrophy [32] or apoptosis [3, 18, 34–36].

Histological data on FSHD muscle sections are scarse, and these descriptions (from either highly affected or not yet/poorly affected muscles) do not cover the large known variability of affected muscles and of patient phenotypes. However, besides the expected DUX4-related atrophic fibers, some publications mention hypertrophic fibers in FSHD muscles (http://neuromuscular.wustl.edu/pathol/fsh.htm; [37]). It was speculated that these were compensatory fibers, but this was never demonstrated [37]. In addition, no pathological mechanism has been suggested to account for other histological observations made in the muscles of patients such as large numbers of splitting and branching myofibrillar bundles, myofibrillar loss and sarcomeric dysfunction, or myonuclear mispositioning, such as clustering of nuclei [20, 28, 38, 39]. Based on the present study, we suggest that these anomalies could be associated with the activation of DUX4c expression, in addition to DUX4 (see also [28]).

In the present study, we used primary cultures of myoblasts derived from FSHD muscle biopsies. In our culture and differentiation conditions (set up by [22]), FSHD myoblasts fuse to myotubes of either atrophic or disorganized (a or dFSHD) phenotypes that might reflect the atrophic and hypertrophic fiber types observed in FSHD muscles. We have previously demonstrated that DUX4 caused the aFSHD myotube phenotype [32], and we now show that it is DUX4c, not DUX4, overexpression that leads to a disorganized myotube phenotype. We could rescue dFSHD myotubes to a normal phenotype with one siRNA targeting the *DUX4c* mRNA. However, additional siRNAs should similarly be tested. Their design is quite difficult because the *DUX4c* gene is very GC rich and nearly identical to *DUX4* in most of the coding sequence; a number of alternatively spliced *DUX4c* mRNAs were detected (Additional file 6: Figure S6B and Ansseau et al. in preparation), but not all of them have been characterized yet. Further studies of the *DUX4c* gene and transcripts will allow the design of additional silencing tools to help characterize DUX4c function in skeletal muscle.

### DUX4c induces misdistribution of myonuclei and cytoskeletal-associated proteins

In previous studies, DUX4c overexpression was shown to result in complete inhibition of the differentiation of TE671 human rhabdomyosarcoma and mouse C2C12 muscle cells [27, 29]. Here, we showed that DUX4c gain of function at different times (before differentiation or after the induction of differentiation) in human healthy primary myoblasts did not affect their capacity to form myotubes even if their differentiation was delayed. However, myotubes with high DUX4c levels appeared hypertrophic and disorganized, with clusters of nuclei. This phenotype is similar to the giant myosacs resulting from the overexpression of skNAC [40, 41]. DUX4c and skNAC share a motif that is essential for interaction with Smyd1 [28]. Both skNAC and Smyd1 play key roles in myofibril assembly [41–43], and excess DUX4c might interfere with their association. In addition, DUX4c overexpression induced an abnormal distribution of troponin T with loss of its normal striation pattern, which could lead to sarcomere malformation and impact contraction, as previously observed following troponin T inhibition [44]. Myoblasts transfected with a backbone vector and maintained in culture more than 6 days after differentiation exhibit spontaneous contractions. However, in a preliminary experiment, we could not observe such contractions in myotubes derived from myoblasts transfected with a DUX4c-expression vector (data not shown). The absence of spontaneous contraction in myotubes with DUX4c overexpression could also partly result from delayed differentiation since myoblasts transfected with a DUX4c expression vector showed a differentiation delay. The impact on troponin T distribution was more severe when DUX4c was expressed before the cytoskeleton reorganized and myofibrils assembled during muscle differentiation. Notably, in FSHD muscles, altered splicing of troponin T mRNA has been observed, which is associated with contraction defects [45]. An abnormal localization of cytoskeletal or cytoskeleton-associated proteins, such as α-tubulin and troponin T, was observed in dFSHD primary myotubes that expressed high levels of endogenous DUX4c as well (Fig. 4, Additional file 4: Figure S4). These cells also exhibited high levels of α-crystallin B chain (CRYAB), a stress-associated chaperone that stabilizes desmin (a DUX4c partner, [28]) and thus maintains intermediate filament integrity; this chaperone is up-regulated in desminopathies as well [46, 47].

Nuclear migration and anchorage have been extensively studied in various cell types, and both the microtubule and actin cytoskeleton networks play major roles in these processes [48–50]. During the skeletal muscle development, thousands of myoblasts fuse together to form multinucleated myotubes, and nuclei progressively migrate to the periphery of the fiber [51–53]. Alterations in the microtubular network and some actin-binding proteins cause changes in the distribution of nuclei along the muscle fiber [50, 53–56]. A lack of desmin also significantly affects the positioning of nuclei [57]. DUX4c overexpression did not induce changes in the protein levels of troponin T but suppressed its normal distribution and striation pattern (Fig. 1). We identified desmin as a DUX4c partner and troponin C and specific actin, myosin, and tubulin isoforms as putative DUX4c partners [28]. We therefore hypothesize that excess DUX4c could interfere with cytoskeleton reorganization in differentiating myotubes, which may result in incorrect localization of myonuclei along the fiber and sarcomere malformations, leading to muscle contraction defects. In agreement with this hypothesis, a previous differential proteomic study showed that proteins displaying an increased relative abundance in dFSHD versus healthy myotubes were mostly associated with the cytoskeleton and particularly with the organization of the microtubule network and myofibril remodeling. This study also demonstrated that the three forms of troponin T exhibited similar abundance in dFSHD and healthy myotubes ([58], Additional file 9: Table S1), as observed in DUX4c-overexpressing myotubes in the present study. In keeping with the lack of a change in expression, no binding site for DUX4 (or, most likely, for DUX4c, as the two proteins share identical homeodomains) was identified in ChIP-seq experiments involving the three genes encoding troponin T isoforms (GEO accession: GSE33838) [14]. In the differential proteomic study, aFSHD myotubes were found to exhibit deregulated cytoskeletal proteins, with an increased abundance of proteins involved in actin cytoskeleton remodeling and a decrease in contractile muscle components ([58], Additional file 9: Table S1). We also observed abnormal troponin T localization in aFSHD myotubes [24], where DUX4 levels are higher [16]. As DUX4 shares common cytoskeletal partners with DUX4c, both proteins could similarly contribute to the observed disorganization [28].

In contrast to the major toxicity exerted by DUX4, DUX4c expression in *Xenopus* embryos was not observed to affect the developing musculature; however, no cytoskeletal analysis was carried out in this study [59]. In a single experiment, we observed actin disorganization in the contractile apparatus of *Ciona intestinalis* following the injection of zygotes with a plasmid allowing muscle-specific expression of green fluorescent protein (GFP) fused to DUX4c, but not to an unrelated protein (collaboration with A. Philips, CRBM, CNRS, Montpellier, Additional file 10: Figure S9).

Abnormal nuclear positioning in muscle diseases is generally only considered as a diagnostic marker; however, it could impact pathological development (reviewed in [50, 60]). We have shown here that DUX4c overexpression induces clusters of nuclei in muscle cell cultures and that dFSHD myotubes present nuclear clusters close to strong DUX4c immunostaining (nuclei and cytoplasm) (Fig. 4, Additional file 4: Figure S4; [28]). In a previous study, we observed DUX4c induction through immunodetection in Western blots in most FSHD muscle biopsies and also in DMD muscle biopsies (at lower levels) in contrast to healthy ones [27]. DUX4c overexpression in FSHD muscles could have an important impact on the development/progression of the pathology that should not be ignored. DUX4c was ruled out as a candidate for FSHD because an FSHD1 family presented a D4Z4 deletion removing one *DUX4c* allele [61, 62]. However, the other DUX4c allele was still present, and DUX4c expression might have occurred but was not analyzed in these patients. Alternatively, the loss of one DUX4c allele could be pathological ([27, 30], and see below). The generation of in vivo models will allow to investigate the impact of DUX4c overexpression in muscle fibers but not its knock-out as there is no DUX4c ortholog in rodents [63]. Other *Dux* genes are present in the mouse (f.i., *Dux* and *Duxbl*), but not in human [63], and *Duxbl* was shown to play roles in myogenesis [64]. Expression of these genes could bias the data obtained in DUX4c transgenic mice in comparison with human muscles. Finally, the study of transgenic models specifically expressing DUX4c in skeletal muscle would require an inducible expression since the highest impact of DUX4c expression in vitro was observed before myofibril formation and the induction of regeneration. We expect that *DUX4c* (located near the D4Z4 locus) could follow a similar kinetics of expression in patient muscle as *DUX4*, the FSHD causal gene; *DUX4* was transiently induced in myoblasts during skeletal muscle regeneration in a mouse model carrying an FSHD D4Z4 genomic fragment [65]. This might be the case since DUX4c was detected in some DMD regenerating fibers [27], is regulated by KLF15, and could be regulated by the *DUX4* myogenic enhancers (see below).

### DUX4c induces β-catenin and other FSHD markers

In addition to nuclear clustering and cytoskeletal disorganization, the DUX4c expression also caused an increase in the content of β-catenin in human myotubes. In healthy proliferating myoblasts, β-catenin is stabilized by WNT signaling and locates to the nucleus, where it activates target genes. When differentiation is induced, β-catenin associates with the M-cadherin complex at the cell membrane. After myotubes have formed, the amount of membrane-associated β-catenin decreases greatly due to Ozz-E3 ubiquitin ligase and proteasome activities [66] as well as that of calpain 3, a calcium-dependent protease that degrades M-cadherin [33]. Interestingly, β-catenin accumulation due to Ozz-E3 inhibition leads to severe defects in myofibrillogenesis, accompanied by an increased frequency of myofibril branching and splitting [66]. Myofibril branching and splitting are also frequently observed in FSHD muscles and have been unexplained until now ([20, 38], Lancelot et al. in preparation). In LGMD2A, a defect in calpain 3 activity leads to hypertrophied myotubes with nuclear accumulation [33]. The clusters of nuclei present in dFSHD and DUX4c-overexpressing myotubes might be associated with simultaneous β-catenin stabilization (Fig. 3a). Canonical WNT/β-catenin was shown as the main coordinator of FSHD-associated signaling and is up-regulated by DUX4 overexpression in murine myoblasts [67]. In addition, DUX4c overexpression in murine myoblasts leads to the accumulation of nuclear beta-catenin [68]. Moreover, we showed that γ-catenin (also known as JUP), which was previously reported as a direct DUX4 target gene [14], was increased in FSHD at the RNA level, although we could not detect protein deregulation. Nuclear clusters have also been observed in FSHD muscle cell cultures selected for their high DUX4 level in a rich culture medium favoring hypertrophy [35]. We hypothesize that these myotubes were co-expressing DUX4c because 4q35 hypomethylation favors activation of both the DUX4 and DUX4c genes [69, 70]. In addition, the choice of culture medium can impact the expression of DUX4 [71] and likely that of DUX4c and might also influence myotube morphology. Accordingly, under our culture conditions, which were identical to those of Barro et al. [22] and differed from those of other studies in FSHD muscle cells, we observed higher levels of DUX4 in aFSHD than in dFSHD myotubes [16] and high DUX4c levels in dFSHD myotubes ([28] and this study).

In primary muscle cell cultures, we have shown that both DUX4c and DUX4 overexpression can induce mu-crystallin (CRYM), as frequently observed in FSHD muscle cells [32, 72]. P53 is up-regulated in FSHD and in DUX4-overexpressing muscle cells [32], but not in DUX4c-overexpressing myoblasts. This observation is in agreement with a comparative analysis of DUX4 and DUX4c following injection of their mRNAs in *Xenopus* embryos [59] and suggests that even if DUX4c (like DUX4) is able to transactivate *PITX1* [4], it does not allow *TP53* up-regulation [73]. Moreover, a recent study suggested that PITX1 transactivation could be due to an indirect effect of DUX4 [74]. DUX4 and DUX4c exhibit both shared and exclusive protein partners [28], and the choice of a given partner could lead to different activities.

DUX4 induces the muscle atrogenes MuRF1 and Atrogin-1, encoding two E3 ubiquitin ligases [32]. Atrogin-1 was not induced by DUX4c overexpression in myotubes after 8 days of differentiation. MuRF1 localizes at the M-line in healthy muscle fibers [75, 76]. However, it is translocated to the nuclei following DUX4 and DUX4c overexpression, as observed in untransfected FSHD muscle cells, and might therefore not induce cytoskeletal protein degradation in the cytoplasm. Nuclear MuRF1 localization has been reported in myocytes in association with a transcription modulator, suggesting a role in gene expression [75], which would be enhanced in FSHD and DUX4- or DUX4c-overexpressing cells.

Here and previous data [32] suggest that the induction of atrophy by DUX4 (but not DUX4c) might instead be Atrogin-1 dependent and would preferentially disrupt Z-line-associated proteins where Atrogin-1 is localized [77]. In addition to desmin, many Z-line proteins as well as proteasomal proteins are putative partners of DUX4/4c [28].

Taken together, these results suggest that DUX4 and DUX4c play a distinct role. In contrast to DUX4, DUX4c does not cause cell death [13, 23, 27, 29] and is implicated in progression to the FSHD disorganized myotube phenotype in our culture conditions, rather than to the atrophic phenotype caused by DUX4 expression [32]. Moreover, the mislocalization of nuclei and the cytoskeletal disorganization observed in FSHD myotubes suggest correlations with other myopathies, such as desminopathies or limb-girdle muscular dystrophy type 2A. In FSHD muscle sections, DUX4c was shown (as in FSHD primary myotubes) in and around abnormal nuclear clusters. Moreover, in FSHD muscle sections, desmin was abnormally localized around these nuclear clusters [28].

Altogether, these data suggest a role for DUX4c in association with the cytoskeleton and the distribution of nuclei during differentiation generating muscle cells with an aberrant number of nuclei in clusters. D.G. Miller’s group [18, 35] suggested that nuclear clustering enhances sensitivity to cell death mediated by DUX4 due to its diffusion in more nuclei and the subsequent amplification of its transcriptional activity. The DUX4 expression is necessary to develop FSHD; however, the very low DUX4 levels detected in healthy muscle cells suggest it is not sufficient [16, 23]. We speculate that DUX4c could be one factor favoring the formation of large nuclear clusters and therefore facilitating the toxic action of DUX4.

On the other hand, DUX4c expression increases during healthy and FSHD myogenic differentiation [27] (as DUX4 in differentiating FSHD myoblasts [78]). During healthy myogenic differentiation, the up-regulation of KLF15 (shown as overexpressed in FSHD muscle cells and biopsies) induces DUX4c, following its binding to and activation of the *D4Z4* enhancer that comes in direct contact with the *DUX4c* promoter in healthy and FSHD myoblasts [69, 70]. KLF15 has been proposed to link myogenic factors (such as MYOD) with *D4Z4* enhancer activity, and their association contributes to the activation of both *DUX4c* and nearby *FRG2* genes during myogenic differentiation and in FSHD muscle cells [70]. In addition, the *DUX4* myogenic enhancers *DME1* and *DME2* might also regulate *DUX4c* expression. Indeed, it was shown that *DME1* and *DME2* enhancers physically interact with the *DUX4* and *FRG2* genes in both healthy and FSHD muscle cells [79]. However, an analysis of the specific primer used to amplify the *DUX4* gene in these 3C chromatin capture experiments indicates that it might also amplify the *DUX4c* gene (Additional file 11: Figure S10). These data reinforce a putative role for DUX4c during myogenic differentiation that might be linked to altered myofibrillogenesis in FSHD primary (atrophic [24] and disorganized) myotubes. We previously reported that DUX4c was expressed (as MYF5) in almost all myoblasts [27, 28] and up-regulated about 1.5- to 2-fold during their differentiation. We also showed that DUX4c localized in healthy myotube cytoplasm around myoblast fusion time. It was also the time when stronger DUX4c staining was observed in and around large clusters of nuclei in FSHD myotubes [28]. We speculate that DUX4c up-regulation and correct intracellular location in healthy myotubes could help myogenesis as suggested by the nature of the DUX4c-induced genes [31]. However further increased DUX4c levels and an incorrect location would perturb myofibrillogenesis as observed in FSHD myotubes. We also previously demonstrated interactions between the DUX4c and MYF5 proteins and suggested DUX4c stabilized MYF5 protein but did not induce its mRNA [27]. If these protein interactions were important during myogenesis, increased MYF5 might rescue the myogenesis defects caused by excess DUX4c as previously reported in mouse myoblast cells [29]. However, if abnormal DUX4c up-regulation occurred after differentiation, MYF5 not being expressed anymore could not counteract DUX4c activity.

### DUX4c gain or loss of function interferes with proliferation

During muscle regeneration, canonical Wnt/β-catenin signaling induces satellite cell proliferation [80], and this pathway is also activated by DUX4c overexpression in myoblasts (see above). We confirmed that DUX4c overexpression induces MYF5 in human primary myoblasts, as previously shown in TE671 and C2C12 cells [27], in agreement with MYF5 induction via the canonical WNT pathway [81, 82]. We previously detected Cyclin A/DUX4c co-immunostaining (S-G2 phases) in DUX4c-overexpressing human TE671 cells as well as induction of PCNA [27]. These cancer cells harbor an activating mutation of N-RAS [83], in contrast to primary myoblasts. In the latter, we only observed a few cells presenting DUX4c staining concomitant with a particular KI67 staining (corresponding either to late G1 or G2 phase, [84]). Other cells with nucleoplasmic KI67 (S or M phases, [84]) did generally not present DUX4c labeling, which suggests incompatibility of DUX4c expression with complete cell cycle progression in primary cells. Alternatively, DUX4c overexpression in transfected cells could induce proliferation of untransfected cells in the vicinity through paracrine regulation. However, our results need to be confirmed in other primary cells.

A lack of Myf5 reduces the myoblast proliferation rate [85, 86]. We have now shown DUX4c inhibition induced myoblast proliferation defects associated with a decrease in KI67 abundance and mitotic events. These results suggest that DUX4c inhibition might affect the proliferation of satellite cells or myoblasts, which are essential for muscle regeneration [87].

A transcriptional study in murine muscle cells showed no effect of DUX4c on proliferative genes in contrast to DUX4 which down-regulated them [31]. However, this could be related to variability among species as shown for DUX4 [31, 88]. Indeed, we previously observed induction of human TE671 but not mouse C2C12 cell proliferation following DUX4c overexpression [27, 29]. Alternatively, DUX4c impact on human myoblast proliferation could be independent of its transcriptional activity, such as direct protein or RNA interactions [28], as previously shown with MYF5 [27], or a paracrine effect resulting from the activation of specific signaling pathways (i.e., the canonical WNT pathway) or both effects.

Taken together, these data indicate that DUX4c could induce proliferation in a WNT-MYF5-dependent manner, which may be prevented by DUX4c inhibition. Therefore, DUX4c might contribute to the FSHD phenotype by interfering with myoblast proliferation in one of two ways: (i) in most patients, DUX4c induction in myoblasts could perturb cell cycle progression or promote it in adjacent cells or (ii) in the few families in which the FSHD deletion has extended to loss of the *DUX4c* gene, there might not be sufficient protein to activate myoblast proliferation.

## Conclusions

In previous studies, we developed antisense oligomers (AOs) and siRNAs targeting *DUX4* mRNA to suppress DUX4 protein expression as a therapeutic approach for FSHD, with a focus on the atrophic myotube phenotype observed in primary cultures [32], and found that *DUX4* mRNA silencing did not correct the disorganized FSHD myotube phenotype [24]. Our gain- and loss-of-function approaches showed that this phenotype was caused by DUX4c expression and suggested that therapeutic strategies for FSHD should take DUX4c into account, in addition to DUX4. Nevertheless, complete DUX4c suppression should not be the goal because a small amount of this protein appears to be required to sustain the myoblast proliferation necessary for muscle regeneration. Other research groups are developing various strategies with the aim of reducing DUX4 expression or toxicity [89–92]. We hypothesize that a precise DUX4/DUX4c ratio might be necessary for therapeutic applications to avoid an imbalance between the atrophic and disorganized myotube morphology. Further studies may better define the interplay between the very similar DUX4/4c proteins and, more precisely, their putative competition for interactions with common partners involved in transcription [14, 93, 94], mRNA processing/regulation/translation [28, 95], or cytoskeletal and myofibril organization [28]. The D4Z4 DNA hypomethylation associated with FSHD causes activation of not only DUX4 but also DUX4c [69, 70]. In contrast to DUX4, which is only immunodetected in 1/1000 to 1/200 of FSHD myonuclei [16, 96], DUX4c is observed in almost all FSHD myoblasts [28], and the present study suggests that DUX4c could initiate muscle fiber disorganization, favor formation of large clusters of nuclei, and disturb regeneration, thereby providing a “weakened” background, facilitating DUX4 toxicity. The levels of both proteins could differ among patients or muscles and during disease progression, and their fluctuating ratios might contribute to disease heterogeneity. Defining DUX4c functions in healthy skeletal muscle should help to design new targeted FSHD therapy by DUX4 or DUX4c inhibition without suppressing DUX4c normal function.

## Additional files

Additional file 1: Figure S1. DUX4c overexpression affects the morphology of human primary myotubes. A. Healthy myoblasts were transfected with the empty vector *pCIneo* or *pCIneo-DUX4c*, and differentiation was induced 24 h after transfection. Six days later, myoblasts were observed under white light (left) before the immunodetection of DUX4c (red) and troponin T (green). B. FSHD myotubes were fixed after 6 days of differentiation. DUX4c and troponin T were detected by immunofluorescence, as described above. Clusters of nuclei are circled and were correlated with troponin T accumulation. Scale bars: 20 μm. (PDF 112 kb)

Additional file 2: Figure S2. DUX4c overexpression induces the formation of ring-like clusters of nuclei and detection of DUX4c in nuclei and the cytoplasm. Healthy primary myoblasts were transfected with *pAC1M2-DUX4c* and differentiated 48 h later. After formation of the first myotubes (day 3), DUX4c expression was induced by the addition of doxycycline (0 ng, 100 ng, or 1 μg) to the culture medium. At day 8, myotubes were fixed. DUX4c (red) and troponin T (green) were detected by immunofluorescence. Nuclei were labeled with DAPI. The circle indicates the cytoplasmic detection of DUX4c at one myotube tip, where troponin T expression was highest in the elongated myotubes. Scale bar: 30 μm. (PDF 89 kb)

Additional file 3: Figure S3. DUX4c overexpression induces β-catenin. Healthy primary myoblasts were transfected with pAC1M2-DUX4c and differentiated 48 h later. After the first myotubes formed (day 3), DUX4c expression was induced by the addition of 100 ng or 1 μg of doxycycline to the culture medium in parallel to the cultures showed in Fig. 2. At day 8, myotubes were fixed and proteins were extracted, separated, transferred to a Western blot, and the indicated proteins were immunodetected as described in Fig. 1b. Histograms: densitometry of the immunoreactive bands normalized to the actin levels (Ponceau red) in each sample. Dox: doxycycline. (PDF 115 kb)

Additional file 4: Figure S4. dFSHD myotubes present abnormal clusters of nuclei overexpressing DUX4c, cytoplasmic DUX4c, and α-tubulin as well as α-crystallin B chain delocalization. A. Healthy and FSHD primary myoblasts were differentiated and fixed 6 days later, and DUX4c (red) and α-tubulin (green) were detected by immunofluorescence. Clusters of nuclei are surrounded by abnormal or almost an absence of α-tubulin. B. The α-crystallin B chain is highly expressed in the cytoplasm of dFSHD myotubes, in contrast to the low nuclear expression observed in healthy myotubes. (PDF 266 kb)

Additional file 5: Figure S5. DUX4c overexpression induces expression of FSHD markers. FSHD and healthy primary myoblasts were transfected with the indicated *pCIneo* expression vectors. A. Total protein extracts were prepared 48 h after transfection. A 30 μg sample of each extract were separated by electrophoresis, transferred to a Western blot, and the indicated proteins immunodetected. This image was used in Vanderplanck et al. (2011) for FSHD, healthy, and DUX4-overexpressing myoblasts. We only added the lane corresponding to DUX4c-overexpressing myoblasts. B. Total protein extracts were prepared 48 h after transfection (top) or 8 days after the induction of differentiation (middle). A 30 μg sample of the extracts was separated via electrophoresis, transferred to a Western blot, and immunodetected. Actin was stained with Ponceau red on the same membrane before immunodetection and was used as the loading control. N.B.: In these conditions, neither endogenous DUX4 nor DUX4c could be immunodetected with MAb 9A12. C. Immunodetection of MuRF1 in healthy FSHD and in DUX4- or DUX4c-overexpressing primary myotubes fixed 7 days after the induction of differentiation. Scale bar: 20 μm. D. γ-Catenin (JUP) mRNA quantification by RT-qPCR in RNA of healthy and FSHD primary muscle cells. The quantity of γ-Catenin mRNA was expressed relative to its amount in healthy cells and set to 1. The means and standard errors are indicated. (PDF 452 kb)

Additional file 6: Figure S6. *DUX4c siRNA* design and efficiency evaluation. A. Schematic representation of the *DUX4c 3′UTR* (positions from Genbank accession number AY500824). The STOP codon, two purine-rich regions which could be used as an alternative 3′end processing (as shown for some histone genes, [97]), the restriction sites *EcoR*I and *Afl*III (used for *p3kb-DUX4c* and *7.5-kb-DUX4c* plasmid constructs, [26]) and the localization of the three designed siRNAs are indicated. Full-length DUX4c transcripts were already described in healthy and FSHD muscle cells [27]. B. Different *DUX4c* RNA ends found following transfection of C2C12 cells with *p7.5-kb-DUX4c* or in primary FSHD myoblasts (indicated by the asterisk). C. Evaluation of DUX4c knock-down using the three siRNAs shown in A. Human muscle TE671 cells were transfected or not (NT) with pCIneo-DUX4c expression vector (DUX4c) and with or without an siRNA targeting DUX4c as indicated (si1, si2, si3). Protein extracts were prepared 3 days later and analyzed by Western blot with the rabbit anti-DUX4c serum. Actin was used as a loading control. The panel with siRNA1 was previously shown in Ansseau et al. 2009 as part of Additional file 3: Figure S3 to confirm anti-DUX4c serum specificity. NT: not transfected. D, E. DUX4c expression in DUX4c-inducible stable TE671 cells [27] transfected with 20 nM *DUX4c siRNA1*. Four hours later, DUX4c expression was either induced or not with 1 μg doxycycline. C. Three or 5 days later, proteins were extracted, and 20 μg were separated on a 10% PAGE-SDS gel and transferred to a Western blot. DUX4c and actin were immunodetected. D. DUX4c detection by immunofluorescence (red) on parallel cultures. (PDF 90 kb)

Additional file 7: Figure S7. siRNA1 specifically silences endogenous *DUX4c*. RNA interference as described in Fig. 6. Endogenous DUX4c was detected by immunofluorescence (red). (PDF 51 kb)

Additional file 8: Figure S8. DUX4c inhibition in myoblasts decreases mitosis. DUX4c inhibition causes cell proliferation defects. FSHD and control primary myoblasts were transfected with siRNAs targeting either the *DUX4* or *DUX4c* mRNA or a non-targeted (*nt*) siRNA. Top: microscope pictures in white light taken of representative fields of each culture. Bottom: The number of mitosis present per field was counted in each culture (10 fields per culture). Histograms show the mean of the mitosis number present per field in each culture. ***p < 0.001 was considered highly significant. (PDF 286 kb)

Additional file 9: Table S1. Cytoskeleton-associated protein quantification in healthy and FSHD myotubes : reorganization of the proteomic data presented separately in Tassin et al (2012: Figure 3, supplemental tables; and unpublished data). Data were subdivided in the three classes of cytoskeletal proteins : actin thin filaments, intermediate filaments and microtubules. In summary, primary healthy, aFSHD (atrophic) and dFSHD (disorganized) myotubes were harvested 4 days after induction of differentiation and protein extracts (TE: total extract; NE: nuclear extract) were analysed by post-digest ICPL coupled to LC-MS/MS. UniProt accession number; Hugo Gene symbol; Protein name; H/L: fold change (v: identified protein without quantification); SD: geometric standard deviation (n.d.: not determined when an abnormal distribution is observed); #: number of peptides used for quantification; *: statistical significance (p<0.05) determined by Student’s t-test. Proteins with an H/L ratio greater than 1.5 are highlighted in red; those with a ratio greater than 1.3 are in pink and those with a ratio greater than 1.2 are in light pink. Proteins with an H/L ratio less than 0.7 are highlighted in green and those with an H/L ratio of 0.7 - 0.8 are highlighted in light green. (PDF 788 kb)

Additional file 10: Figure S9. DUX4c expression affects actin in the muscle cells of *Ciona intestinalis* zygotes. Injection of a plasmid expressing GFP-DUX4c or GFP fused to an unrelated protein under a muscle-specific promoter in *Ciona intestinalis* zygotes led to actin (phalloidin staining) disorganization in the contractile apparatus (collaboration with A. Philips, CRBM, CNRS, Montpellier). (PDF 122 kb)

Additional file 11: Figure S10. DUX4 myogenic enhancer (DME1 and 2) might interact with the DUX4c gene. The DUX4 forward primer used in a 3C capture experiment [79] is highlighted in a common region in *DUX4* and *DUX4c* ORF sharing 100% identity (Accession numbers AF AF117653 and AY500824). In Himeda et al. [79], RT-qPCR was performed using this primer in combination with either DIR1 (containing DME1) or DIR2 (containing DME2) primer to confirm DUX4-DME1 or DUX4-DME2 interactions. The *Blg*II site (boxed) used to digest chromatin-linked DNA regions is also shown and is located 226-bp downstream of the 5’end of the *DUX4* primer. The region between DUX4 primer and the *Bgl*II site share 100% identity. (PDF 287 kb)

## Abbreviations

aFSHD: Atrophic FSHD
CRYAB: Alpha-crystallin B chain
CRYM: Ketimine reductase mu-crystallin
dFSHD: Disorganized FSHD
DMD: Duchenne muscular dystrophy
DNMT3B: DNA (cytosine-5)-methyltransferase 3B
DUX4: Double homeobox 4
DUX4c: Double homeobox 4 centromeric
FSHD: Facioscapulohumeral dystrophy
GFP: Green fluorescent protein
JUP: Junction plakoglobin
KI67: Proliferation marker protein Ki-67
KLF15: Krüppel-like transcription factor 15
LGMD2A: Limb-girdle muscular dystrophy type 2A
MAFbx: Muscle atrophy F-box protein
mRNA: Messenger ribonucleic acid
MuRF1: Muscle-specific RING finger protein 1
MYF5/Myf5: Human/mouse myogenic factor 5
nt: Non-targeted
PCNA: Proliferating cell nuclear antigen
PITX1: Pituitary homeobox 1
siRNA: Silencing RNA
SkNAC: Skeletal nascent polypeptide-associated complex alpha, a muscle-specific isoform of Naca
SMCHD1: Structural maintenance of chromosomes flexible hinge domain-containing protein 1
SMYD: SET and MYND domain-containing protein 1
SNP: Single nucleotide polymorphism
TP53/P53: Gene/protein cellular tumor antigen p53
WNT: Wingless-related integration site
