# Supplementary figures and images for: Overexpression of the double homeodomain protein DUX4c interferes with myofibrillogenesis and induces clustering of myonuclei
Source: Skelet Muscle. 2018 Jan 12;8:2. doi: 10.1186/s13395-017-0148-4 (PMC5767009; doi:10.1186/s13395-017-0148-4)

**A.**

Healthy myotubes

pCI

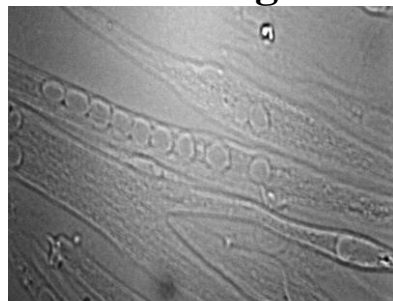

DUX4c

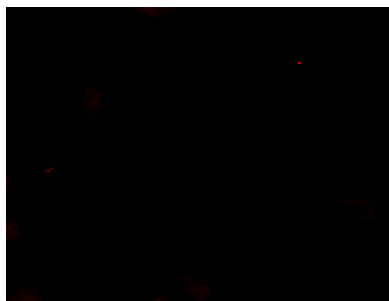

Troponin T

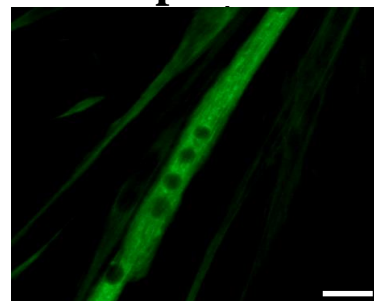

DUX4c

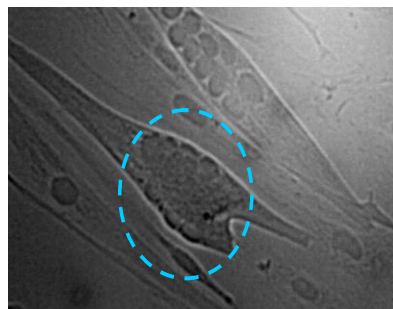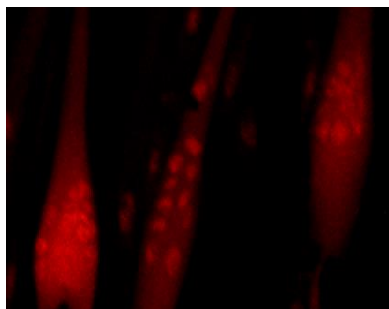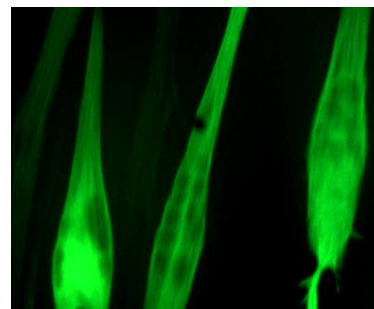**B.**

FSHD myotubes

Troponin T

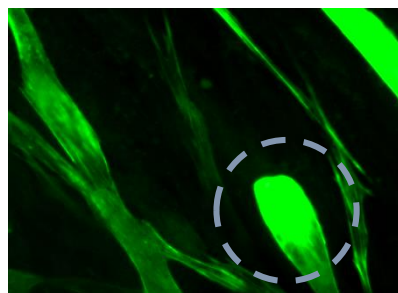

DUX4c

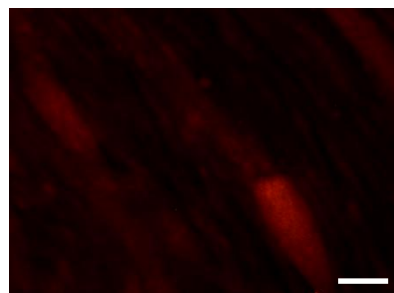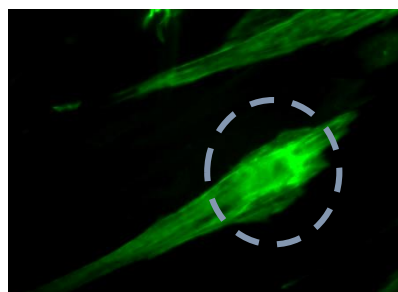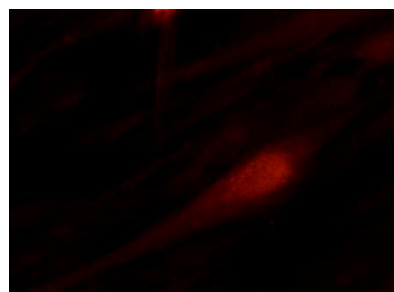

Supplement: Supplementary file 1 — DUX4c overexpression affects the morphology of human primary myotubes. A. Healthy myoblasts were transfected with the empty vector pCIneo or pCIneo-DUX4c, and differentiation was induced 24 h after transfection. Six days later, myoblasts were observed under white light (left) before the immunodetection of DUX4c (red) and troponin T (green). B. FSHD myotubes were fixed after 6 days of differentiation. DUX4c and troponin T were detected by immunofluorescence, as described above. Clusters of nuclei are circled and were correlated with troponin T accumulation. Scale bars: 20 μm. (PDF 112 kb) [file 13395_2017_148_MOESM1_ESM.pdf]

Figure S2

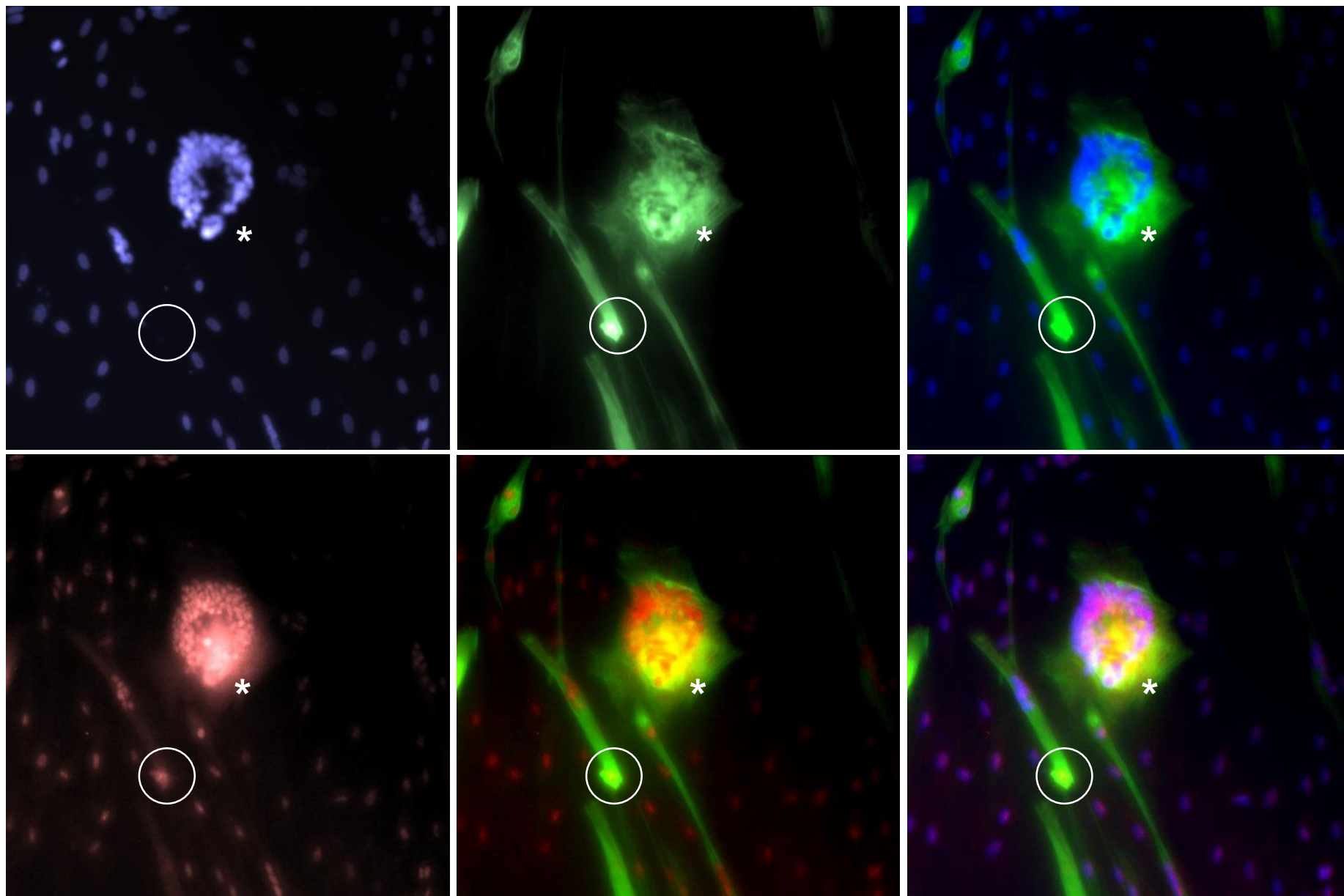

Supplement: Supplementary file 2 — DUX4c overexpression induces the formation of ring-like clusters of nuclei and detection of DUX4c in nuclei and the cytoplasm. Healthy primary myoblasts were transfected with pAC1M2-DUX4c and differentiated 48 h later. After formation of the first myotubes (day 3), DUX4c expression was induced by the addition of doxycycline (0 ng, 100 ng, or 1 μg) to the culture medium. At day 8, myotubes were fixed. DUX4c (red) and troponin T (green) were detected by immunofluorescence. Nuclei were labeled with DAPI. The circle indicates the cytoplasmic detection of DUX4c at one myotube tip, where troponin T expression was highest in the elongated myotubes. Scale bar: 30 μm. (PDF 89 kb) [file 13395_2017_148_MOESM2_ESM.pdf]

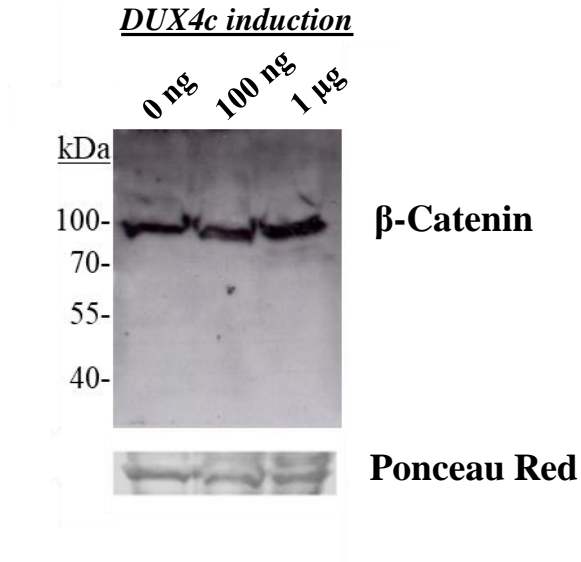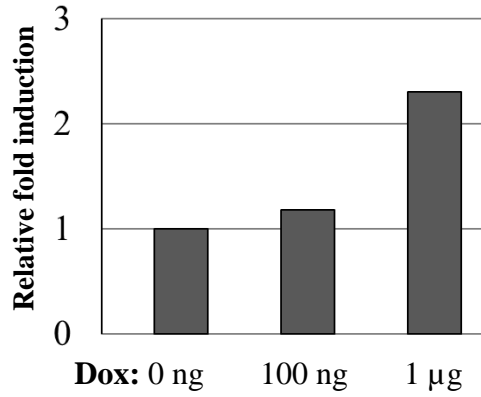

Supplement: Supplementary file 3 — DUX4c overexpression induces β-catenin. Healthy primary myoblasts were transfected with pAC1M2-DUX4c and differentiated 48 h later. After the first myotubes formed (day 3), DUX4c expression was induced by the addition of 100 ng or 1 μg of doxycycline to the culture medium in parallel to the cultures showed in Fig. 2. At day 8, myotubes were fixed and proteins were extracted, separated, transferred to a Western blot, and the indicated proteins were immunodetected as described in Fig. 1b. Histograms: densitometry of the immunoreactive bands normalized to the actin levels (Ponceau red) in each sample. Dox: doxycycline. (PDF 115 kb) [file 13395_2017_148_MOESM3_ESM.pdf]

Figure S4

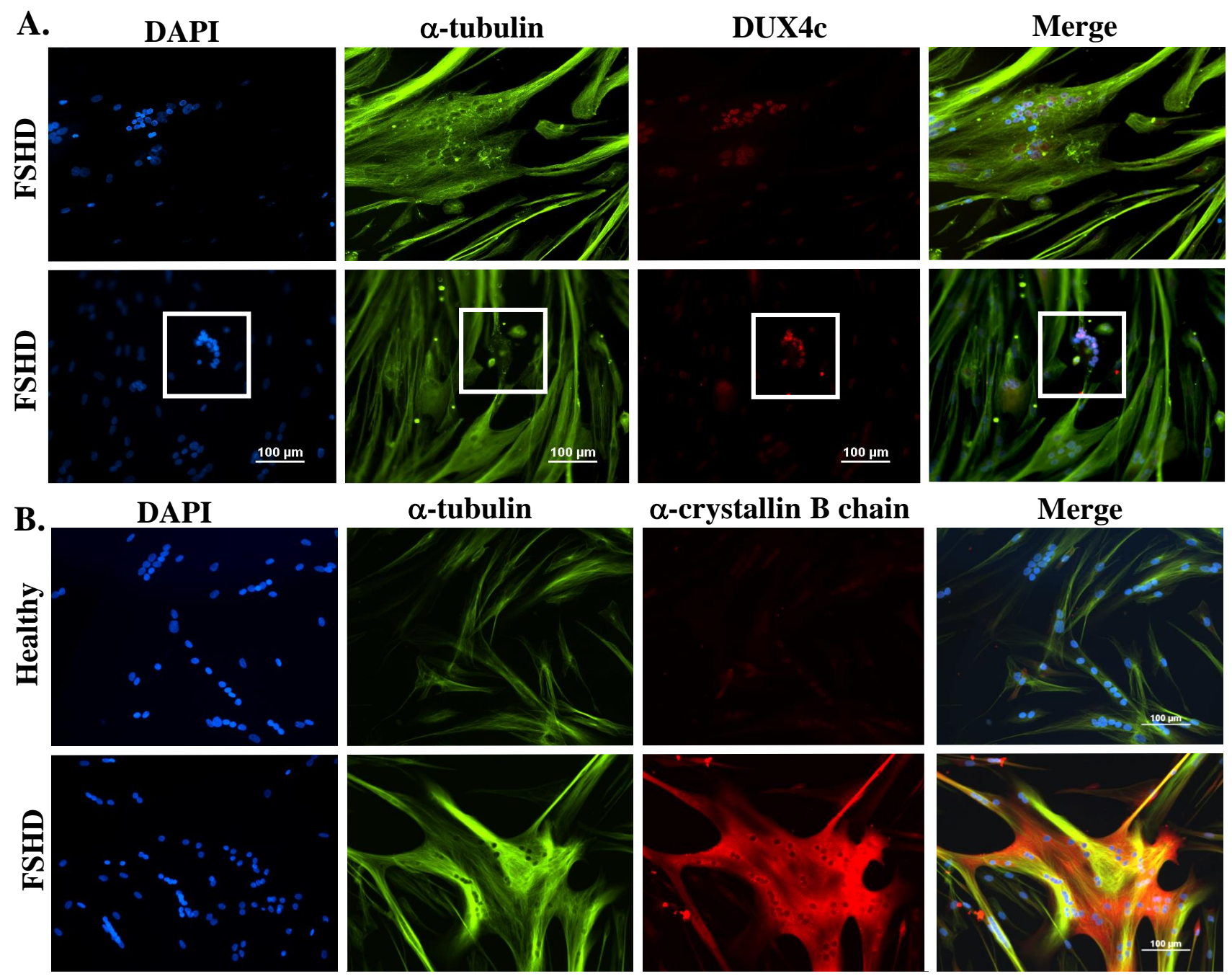

Supplement: Supplementary file 4 — dFSHD myotubes present abnormal clusters of nuclei overexpressing DUX4c, cytoplasmic DUX4c, and α-tubulin as well as α-crystallin B chain delocalization. A. Healthy and FSHD primary myoblasts were differentiated and fixed 6 days later, and DUX4c (red) and α-tubulin (green) were detected by immunofluorescence. Clusters of nuclei are surrounded by abnormal or almost an absence of α-tubulin. B. The α-crystallin B chain is highly expressed in the cytoplasm of dFSHD myotubes, in contrast to the low nuclear expression observed in healthy myotubes. (PDF 266 kb) [file 13395_2017_148_MOESM4_ESM.pdf]

**A.**

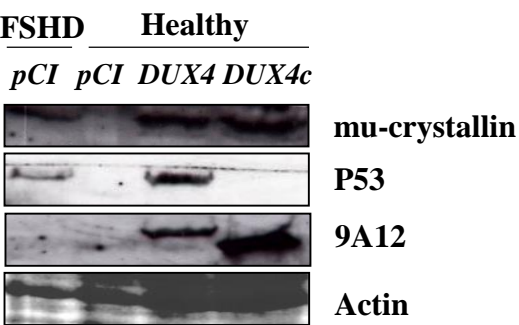

**B.**

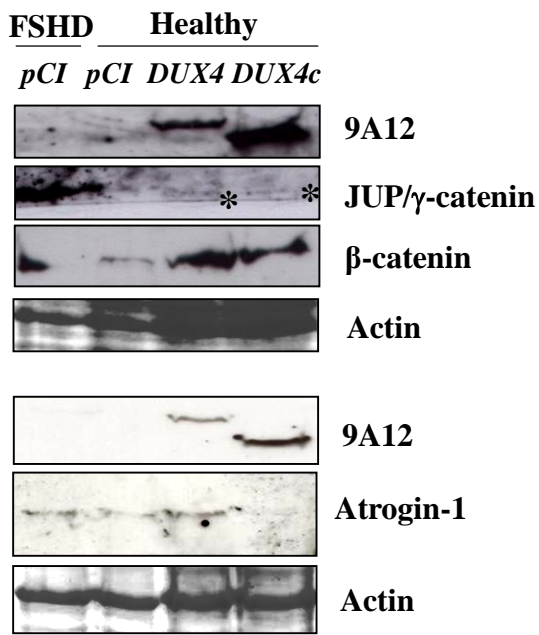

**C.**

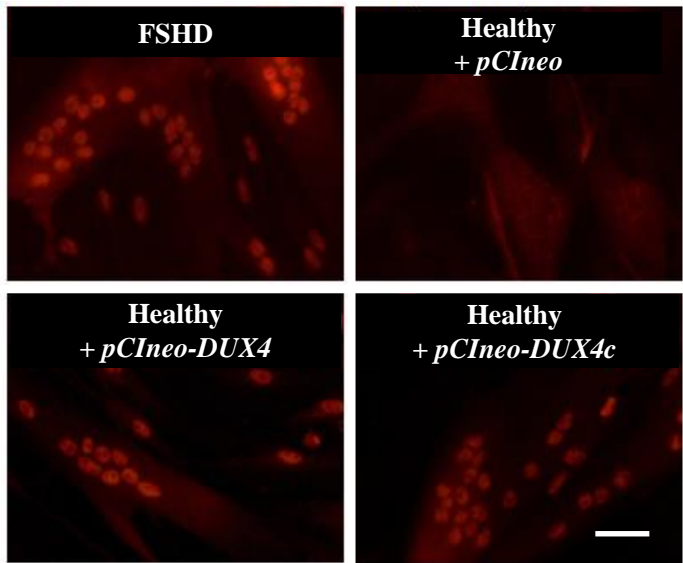

**D.**

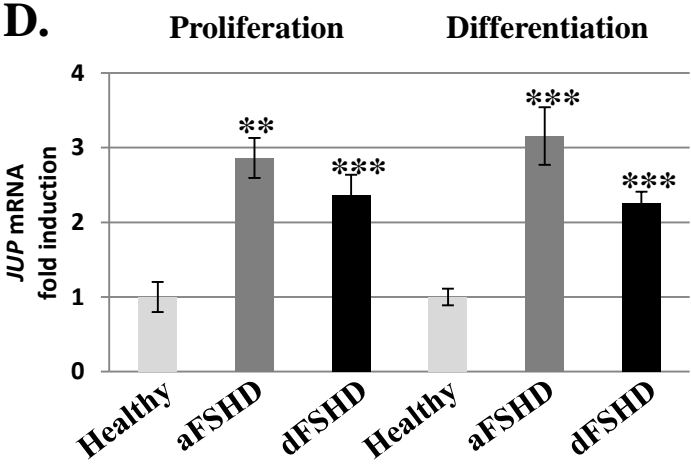

Supplement: Supplementary file 5 — DUX4c overexpression induces expression of FSHD markers. FSHD and healthy primary myoblasts were transfected with the indicated pCIneo expression vectors. A. Total protein extracts were prepared 48 h after transfection. A 30 μg sample of each extract were separated by electrophoresis, transferred to a Western blot, and the indicated proteins immunodetected. This image was used in Vanderplanck et al. (2011) for FSHD, healthy, and DUX4-overexpressing myoblasts. We only added the lane corresponding to DUX4c-overexpressing myoblasts. B. Total protein extracts were prepared 48 h after transfection (top) or 8 days after the induction of differentiation (middle). A 30 μg sample of the extracts was separated via electrophoresis, transferred to a Western blot, and immunodetected. Actin was stained with Ponceau red on the same membrane before immunodetection and was used as the loading control. N.B.: In these conditions, neither endogenous DUX4 nor DUX4c could be immunodetected with MAb 9A12. C. Immunodetection of MuRF1 in healthy FSHD and in DUX4- or DUX4c-overexpressing primary myotubes fixed 7 days after the induction of differentiation. Scale bar: 20 μm. D. γ-Catenin (JUP) mRNA quantification by RT-qPCR in RNA of healthy and FSHD primary muscle cells. The quantity of γ-Catenin mRNA was expressed relative to its amount in healthy cells and set to 1. The means and standard errors are indicated. (PDF 452 kb) [file 13395_2017_148_MOESM5_ESM.pdf]

Figure S6

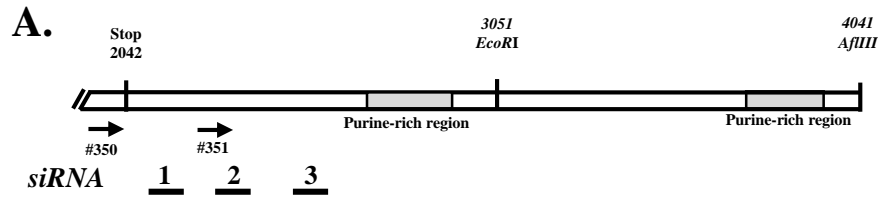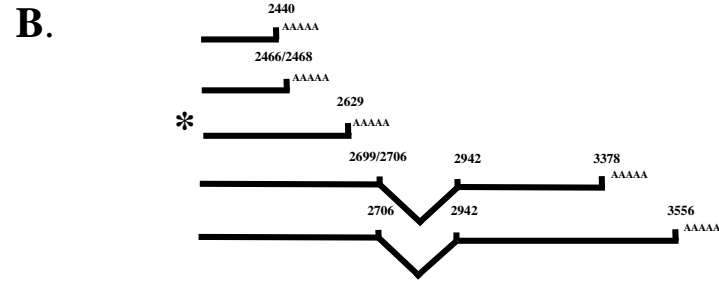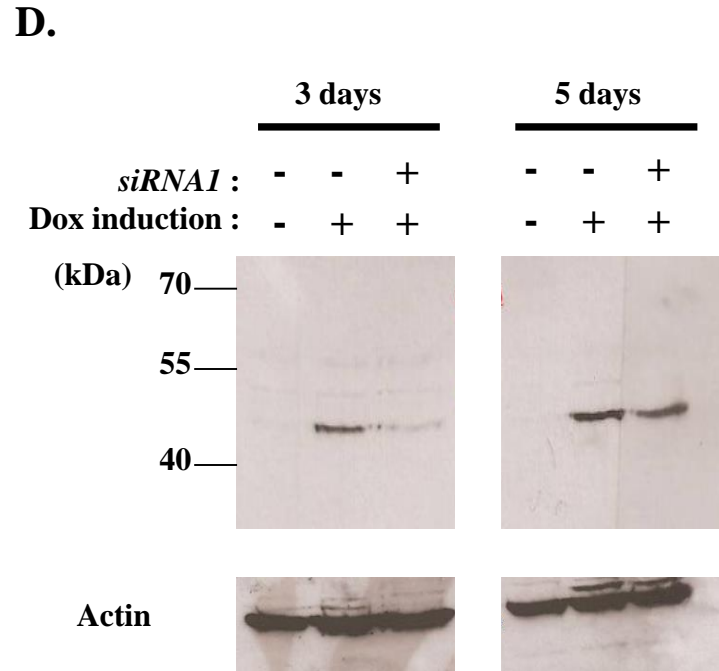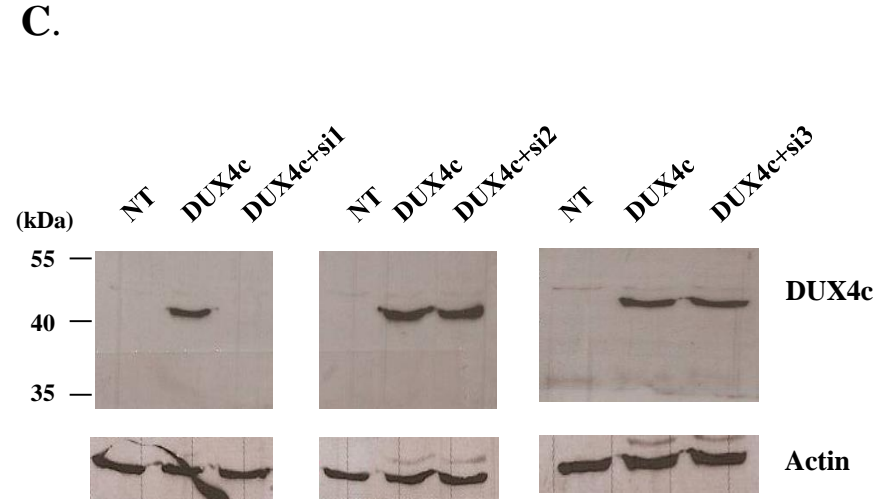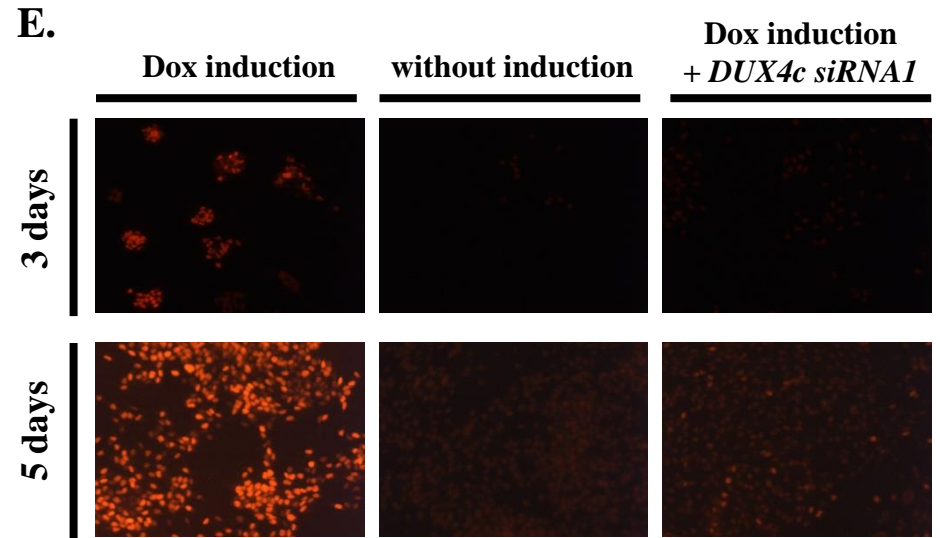

Supplement: Supplementary file 6 — DUX4c siRNA design and efficiency evaluation. A. Schematic representation of the DUX4c 3′UTR (positions from Genbank accession number AY500824). The STOP codon, two purine-rich regions which could be used as an alternative 3′end processing (as shown for some histone genes, [97]), the restriction sites EcoRI and AflIII (used for p3kb-DUX4c and 7.5-kb-DUX4c plasmid constructs, [26]) and the localization of the three designed siRNAs are indicated. Full-length DUX4c transcripts were already described in healthy and FSHD muscle cells [27]. B. Different DUX4c RNA ends found following transfection of C2C12 cells with p7.5-kb-DUX4c or in primary FSHD myoblasts (indicated by the asterisk). C. Evaluation of DUX4c knock-down using the three siRNAs shown in A. Human muscle TE671 cells were transfected or not (NT) with pCIneo-DUX4c expression vector (DUX4c) and with or without an siRNA targeting DUX4c as indicated (si1, si2, si3). Protein extracts were prepared 3 days later and analyzed by Western blot with the rabbit anti-DUX4c serum. Actin was used as a loading control. The panel with siRNA1 was previously shown in Ansseau et al. 2009 as part of Additional file 3: Figure S3 to confirm anti-DUX4c serum specificity. NT: not transfected. D, E. DUX4c expression in DUX4c-inducible stable TE671 cells [27] transfected with 20 nM DUX4c siRNA1. Four hours later, DUX4c expression was either induced or not with 1 μg doxycycline. C. Three or 5 days later, proteins were extracted, and 20 μg were separated on a 10% PAGE-SDS gel and transferred to a Western blot. DUX4c and actin were immunodetected. D. DUX4c detection by immunofluorescence (red) on parallel cultures. (PDF 90 kb) [file 13395_2017_148_MOESM6_ESM.pdf]

Figure S7

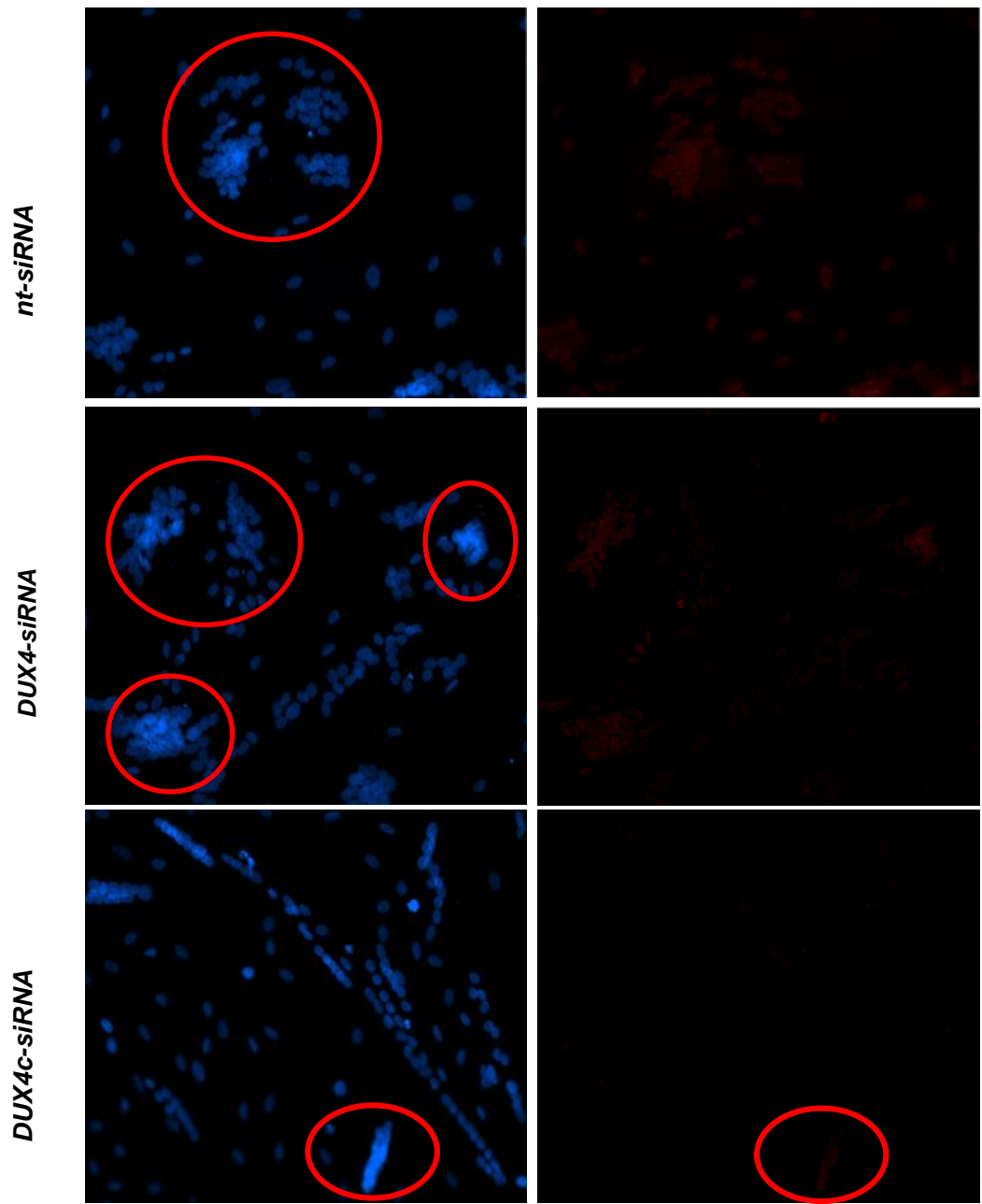

Supplement: Supplementary file 7 — siRNA1 specifically silences endogenous DUX4c. RNA interference as described in Fig. 6. Endogenous DUX4c was detected by immunofluorescence (red). (PDF 51 kb) [file 13395_2017_148_MOESM7_ESM.pdf]

Human primary myoblasts in proliferation

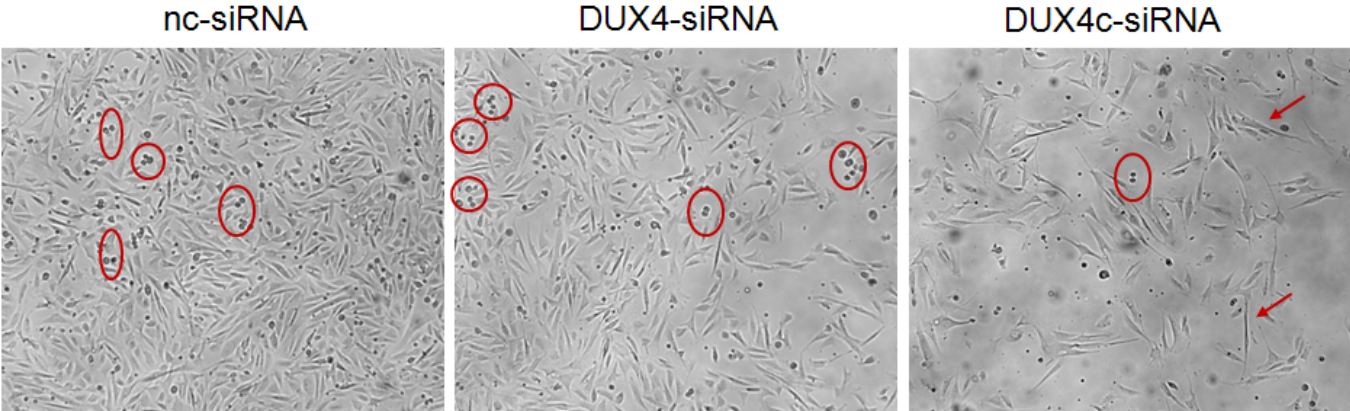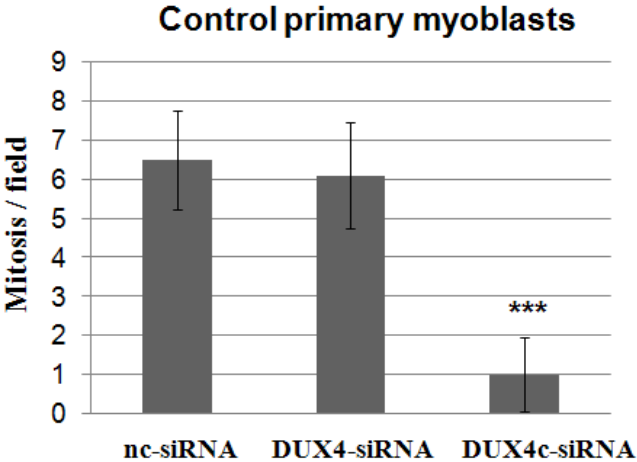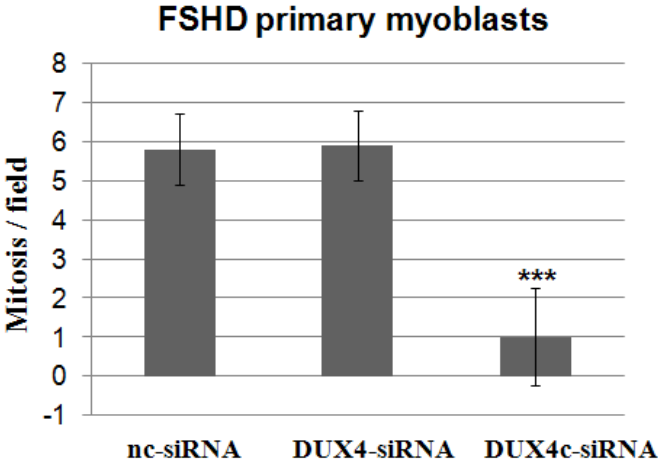

Supplement: Supplementary file 8 — DUX4c inhibition in myoblasts decreases mitosis. DUX4c inhibition causes cell proliferation defects. FSHD and control primary myoblasts were transfected with siRNAs targeting either the DUX4 or DUX4c mRNA or a non-targeted (nt) siRNA. Top: microscope pictures in white light taken of representative fields of each culture. Bottom: The number of mitosis present per field was counted in each culture (10 fields per culture). Histograms show the mean of the mitosis number present per field in each culture. ***p < 0.001 was considered highly significant. (PDF 286 kb) [file 13395_2017_148_MOESM8_ESM.pdf]

*Transfection: GFP-DUX4c*

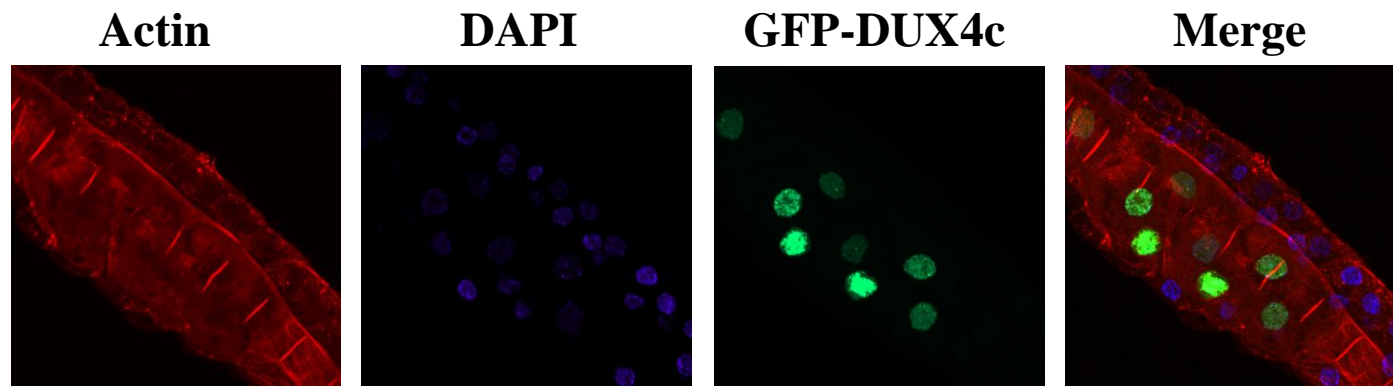

*Transfection: GFP (Control)*

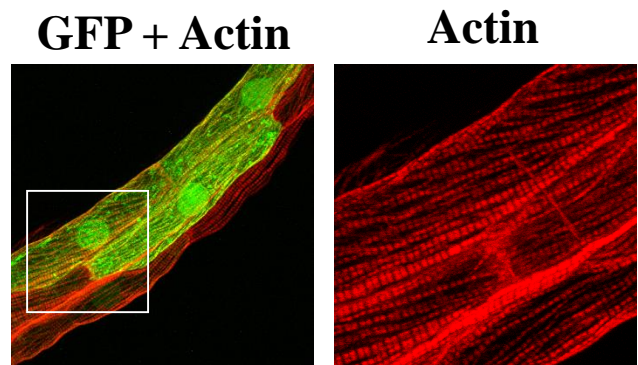

Supplement: Supplementary file 10 — DUX4c expression affects actin in the muscle cells of Ciona intestinalis zygotes. Injection of a plasmid expressing GFP-DUX4c or GFP fused to an unrelated protein under a muscle-specific promoter in Ciona intestinalis zygotes led to actin (phalloidin staining) disorganization in the contractile apparatus (collaboration with A. Philips, CRBM, CNRS, Montpellier). (PDF 122 kb) [file 13395_2017_148_MOESM10_ESM.pdf]
