# Supplementary material for: Overexpression of the double homeodomain protein DUX4c interferes with myofibrillogenesis and induces clustering of myonuclei
Source: Skelet Muscle. 2018 Jan 12;8:2. doi: 10.1186/s13395-017-0148-4 (PMC5767009; doi:10.1186/s13395-017-0148-4)
Supplement: Supplementary file 9 — Cytoskeleton-associated protein quantification in healthy and FSHD myotubes : reorganization of the proteomic data presented separately in Tassin et al (2012: Figure 3, supplemental tables; and unpublished data). Data were subdivided in the three classes of cytoskeletal proteins : actin thin filaments, intermediate filaments and microtubules. In summary, primary healthy, aFSHD (atrophic) and dFSHD (disorganized) myotubes were harvested 4 days after induction of differentiation and protein extracts (TE: total extract; NE: nuclear extract) were analysed by post-digest ICPL coupled to LC-MS/MS. UniProt accession number; Hugo Gene symbol; Protein name; H/L: fold change (v: identified protein without quantification); SD: geometric standard deviation (n.d.: not determined when an abnormal distribution is observed); #: number of peptides used for quantification; *: statistical significance (p<0.05) determined by Student’s t-test. Proteins with an H/L ratio greater than 1.5 are highlighted in red; those with a ratio greater than 1.3 are in pink and those with a ratio greater than 1.2 are in light pink. Proteins with an H/L ratio less than 0.7 are highlighted in green and those with an H/L ratio of 0.7 - 0.8 are highlighted in light green. (PDF 788 kb) [file 13395_2017_148_MOESM9_ESM.pdf]

S1 Table

			dFSHD12_TE				dFSHD12_NE				aFSHD51_TE				aFSHD51_NE			
Accession	Gene	Protein	H/L	SD	#	bold	H/L	SD	#	bold	H/L	SD	#	bold	H/L	SD	#	bold
Intermediate filament (or associated proteins)																		
P17661	DES	Desmin	0.91	N.D.	37		1.06	1.35	19		0.89	1,13	33	*	1.20	1.21	17	*
P02545	LMNA	Prelamin-A/C	0.90	1.25	30	*	1.07	1.26	20		0.96	1.23	34		1.21	1.24	19	*
P48681	NES	Nestin	0.91	N.D.	60		0.94	1.25	27		0.72	1.20	50	*	0.89	1.27	31	*
P08670	VIM	Vimentin	1.04	N.D.	35		1.24	1.24	14	*	1.21	1.18	36	*	1.39	1.17	16	*
Q15149	PLEC	Plectin-1	1.10	1.28	19		1.05	1.10	3		1.07	1.23	26		1.25	1.27	7	*
P02511	CRYAB	Alpha-crystallin B chain (HspB5)	1.47	1.17	2		1.17	2			1.14	1.12	2					
Tubulin (or associated proteins)																		
P62158	CALM1	Calmodulin (CaM)	0.83	0.00	1		✓				0.93	0.00	1					
Q8WUM4	PDCD6IP	Programmed cell death 6-interacting protein	1.34	0.00	1													
Q71U36	TUBA1A	Tubulin α-1A chain (α-tubulin 3)	1.44	1.12	3	*												
P07437	TUBB	Tubulin β chain (Tubulin β-5 chain)	1.52	0.00	1						✓							
Q14980	NUMA1	Nuclear mitotic apparatus protein 1					0.68	0.00	1						0.75	0.00	1	
P27816	MAP4	Microtubule-associated protein 4					✓				1.10		1					
P78559	MAP1A	Microtubule-associated protein 1A					0.76	0.00	1									
Q6PEY2	TUBA3E	Tubulin α-3E chain (α-tubulin 3E)					0.91	0.00	1		1.12	0.00	1		0.98	1.12	3	
P68371	TUBB2C	Tubulin β-2C chain (Tubulin β-2 chain)					0.93	1.13	7									
Q3ZCM7	TUBB8	Tubulin β-8 chain					0.97	1.09	4									
P34897	SHMT2	Serine hydroxymethyltransferase (serine methylase)	0.93	0.00	1													
Q07065	CKAP4	Cytoskeleton-associated protein 4 (p63)	0.98	1.24	5		0.96	0.00	1		1.08	1.28	6		0.75	0.00	1	
Q66GS9	CEP135	Centrosomal protein of 135 kDa (Centrosomal protein 4)	0.84	0.00	1													

Q96AQ6	PBXIP1	Pre-B cell leukemia transcription factor-interacting protein 1	0.74	0.00	1						0.80		1					
P17897	TCP1	T-complex protein 1 subunit alpha (CCT-alpha)					0.84	0.00	1									
Q13409	DYNC1I2	Cytoplasmic dynein intermediate chain 2 (DH IC-2)					1.01	0.00	1									
Q8TD57	DNAH3	Dynein heavy chain 3 (Dnahc3-b)									✓							
Q9UPN3	MACF1	Microtubule-actin cross- linking factor 1 (Trabeculin-alpha)					✓											
Actin (or associated including myofibril-associated porteins)																		
P60709	ACTB	Actin, cytoplasmic 1 (β-actin)	1.11	N.D.	38		1.41	1.43	21	*	1.13	1.18	35	*	1.22	1.17	22	*
Q562R1	ACTBL2	β-actin-like protein 2 (Kappa-actin)	1.03	1.17	17						1.15	N.D.	17					
P62736	ACTA2	Actin, aortic smooth muscle (α-actin-2)	1.04	N.D.	39						1.02	1.29	40					
P68032	ACTC1	Actin, alpha cardiac muscle 1	1.02	N.D.	40		1.39	1.43	25	*	1.02	1.29	40		1.13	1.22	26	*
Q9BYX7	ACTBM	Putative Beta-actin-like protein 3 (Kappa actin)									1.12	1.07	5	*				
P12814	ACTN1	α-actinin-1	0.94	1.24	29		1.03	1.32	22		1.04	1.20	28		1.13	1.27	20	*
P35609	ACTN2	α-actinin-2	0.89	1.18	14	-	0.97	1.33	16		0.93	1.29	15		1.01	1.25	13	
O43707	ACTN4	α-actinin-4	0.87	1.20	28	*	1.01	1.24	20		1.11	1.19	27	*	1.18	1.26	19	*
Q53GG5	PDLIM3	PDZ and LIM domain protein 3 (Actinin-associated LIM protein)					0.63	0.00	1		1.00	1.32	3		1.07	0.00	1	
Q9NR12	PDLIM7	PDZ and LIM domain protein 7 (Actinin-associated LIM protein)	0.73	0.00	1		0.81	1.13	2						1.11	1.16	2	
Q9UHB6	LIMA1	LIM domain and actin-binding protein 1					0.92	0.00	1		✓				1.33	1.12	2	
O75112	LDB3	LIM domain-binding protein 3 (protein cypher)					0.90	1.11	2									
Q9Y490	TLN1	Talin-1	✓				✓								0.97	1.08	2	
Q13813	SPTAN1	Spectrin α chain, brain 1	1.03	1.22	9		0.89	1.28	9		0.74	1.11	9	*	0.71	1.14	6	*
Q01082	SPTBN1	Spectrin β chain, brain 1	0.89	0.00	1		1.24	1.09	4	*	0.77	1.16	6	*	0.98	0.00	1	
P60981	DSTN	Destrin (Actin-depolymerizing factor)	1.25	0.00	1						1.12		1					
Q15511	ARPC5	Actin-related protein 2/3 complex subunit 5 (p16-ARC)													✓			

Q9BX66	SORBS1	Sorbin and SH3 domain-containing protein 1 (CAP)					0.80	0.00	1									
O94875	SORBS2	Sorbin and SH3 domain-containing protein 2 (ArgBP2)	✓				0.93	0.00	1		0.78	1.20	2					
Q16643	DBN1	Drebrin	0.59	0.00	1		✓				0.91	1.23	5		1.19	0.00	1	
P60981	DSTN	Destrin (ADF)	1.25	0.00	1						1.12		1					
Q9UMS6	SYNPO	Synaptopodin-2 (Myopodin	1.02	N.D.	6		1.14	1.31	5		0.82	1.30	6		0.97	1.20	4	
Q9H987	SYNPO2L	Synaptopodin 2-like protein					0.75	1.08	2						0.67	1.19	2	
Q9ULV4	CORO1C	Coronin-1C (Coronin-3)	✓				0.80	1.03	2		✓				1.34	1.17	2	
P21333	FLNA	Filamin-A	1.02	1.29	31		1.00	1.34	16		1.46	1.21	40	*	1.49	1.23	21	*
O75369	FLNB	Filamin-B									✓							
Q14315	FLNC	Filamin-C	1.09	1.24	28		1.07	1.30	20		1.14	1.22	33	*	0.99	1.22	14	
P63000	RAC1	Ras-related C3 botulinum toxin substrate 1	✓								0.9		1					
P31943	HNRNPH1	Heterogeneous nuclear ribonucleoprotein H (hnRNP H)	0.95	1.19	4		1.01	1.20	2		0.87	1.03	3	*				
P55795	HNRNPH2	Heterogeneous nuclear ribonucleoprotein H2 (hnRNP H2)					0.89	0.00	1		1.01	0.00	1					
P04075	ALDOA	Fructose-bisphosphate aldolase A (Muscle type aldolase)	1.09	0.00	1		0.99	0.00	1		1.03	1.19	2					
P06396	GSN	Gelsolin (AGEL)									1.23		1					
Q15942	ZYX	Zyxin					1.03	0.00	1									
Q16181	SEPT7	Septin-7 (CDC10 protein homolog)					✓											
Q9UHD8	SEPT9	Septin-9 (Septin D1)					1.02	0.00	1									
Q9VA7	SEPT11	Septin-11					1.23	0.00							0.99	0.00	1	
Q15654	TRIP6	Thyroid receptor-interacting protein 6	1.00	0.00	1		0.77	0.00										
P06748	NPM1	Nucleophosmin	0.86	1.13	5	*	1.03	1.09	3		0.81	1.20	5		1.05	1.24	2	
P07355	ANXA2	Annexin A2	1.16	N.D.	17		✓				1.10	1.34	19		0.89	1.19	2	
P18206	VCL	Vinculin (metavinculin)									✓				1.29	0.00	1	
P23528	CFL1	Cofilin-1 (p18)									1.10		1					
P40123	CAP2	Adenylyl cyclase associated protein 2	1.07	0.00	1													

Q01995	TAGLN	Transgelin (SM22-alpha)									✓							
Q8WZ42	TTN	Titin	0.84	0.00	1		1.02	1.32	31		0.76	1.08	4	*	0.71	1.18	8	*
P07951	TPM2	Tropomyosin β chain (Tropomyosin-2)	1.12	1.23	16	*	1.09	1.27	8		0.95	1.15	14		1.16	1.16	4	
P09493	TPM1	Tropomyosin α-1 chain (Tropomyosin-1)	1.13	1.24	9		1.05	1.30	6						1.19	1.15	5	*
P06753	TPM3	Tropomyosin α-3 chain (Tropomyosin-3)	1.02	1.26	5						0.96	1.12	7					
P67936	TPM4	Tropomyosin α-4 chain (Tropomyosin-4)									1.00	1.10	6		1.28	1.08	2	
Q6WCQ1	MPRIP	Myosin phosphatase Rho-interacting protein					0.73	1.27	3		1.19		1		✓			
P29966	MARCKS	Myristoylated alanine-rich C-kinase substrate	1.15	1.24	2						1.20	1.16	3					
Q0ZGT2	NEXN	Nexilin	✓				✓				0.98	1.11	2		1.06	1.16	3	
P63316	TNNC1	Troponin C, slow skeletal and cardiac muscles	0.93	0.00	1		0.84	0.00	1		0.83		1		✓			
P19237	TNNI1	Troponin I, slow skeletal muscle	1.09	1.21	8		0.95	1.28	5		0.81	1.28	8	*	0.83	1.23	4	
P45379	TNNT2	Troponin T, cardiac muscle (TnTc)	1.03	1.19	5		1.23	1.25	5		0.77	1.19	5	*	0.96	1.17	5	
P45378	TNNT3	Troponin T, fast skeletal muscle (TnTf)									0.84		1		✓			
Q15417	CNN3	Calponin-3	1.29	0.00	1													
Q05682	CALD1	Caldesmon (CDM)	1.16	1.18	5		1.06	1.29	9		1.60	1.24	3		1.54	1.26	11	*
Q12797	ASPH	Aspartyl/asparaginyl beta-hydroxylase									0.73		1					
Q13884	SNTB1	Beta-1 syntrophin (Syntrophin-2)	1.08	0.00	1													
Q15149	PLEC	Plectin (PLTN)	1.10	1.28	19		1.05	1.10	3		1.07	1.23	26		1.25	1.27	7	*
P26038	MSN	Moesin	0.97	1.10	4						0.80		1		0.78	0.00	1	
Q702N8	XIRP1	Xin actin-binding repeat containing protein 1	0.92	1.17	9		0.95	1.21	15		0.75	1.19	10	*	1.02	1.15	10	
Q96IZ0	PAWR	PRKC apoptosis WT1 regulator protein					0.92	0.00	1		✓				1.97	0.00	1	
Q9NR64	KLHL1	Kelch-like protein 1					✓											
O60662	KBTBD10	Kelch-related protein 1	✓												✓			

Q9Y696	CLIC4	Chloride intracellular channel protein 4	1.04	1.08	2		0.96	0.00	1		1.32		1					
P80723	BASP1	Brain acid soluble protein 1 (NAP-22)					0.69	1.22	3		0.89	1.12	2					
Q8WX93	PALLD	Palladin					1.16	1.13	5									
Q86TC9	MYPN	Myopalladin									✓							
Myosin isoforms	Tassin et al 2012 (Figure 4) : in summary, the skeletal muscle myosin isoforms were decreased in aFSHD myotubes while non-muscle myosin complexes were more abundant. By contrast, myosin isoforms were not reduced in FSHD disorganized myotubes.																	

Cytoskeleton-associated protein quantification in healthy and FSHD myotubes : reorganization of the proteomic data presented separately in Tassin et al (2012: Figure 3, supplemental tables; and unpublished data). Data were subdivided in the three classes of cytoskeletal proteins : actin thin filaments, intermediate filaments and microtubules. In resume, primary healthy, aFSHD (atrophic) and dFSHD (disorganized) myotubes were harvested 4 days after induction of differentiation and protein extracts (TE: total extract; NE: nuclear extract) were analysed by post-digest ICPL coupled to LC-MS/MS. UniProt accession number; Hugo Gene symbol; Protein name; H/L: fold change (✓: identified protein without quantification); SD: geometric standard deviation (n.d.: not determined when an abnormal distribution is observed); #: number of peptides used for quantification; *: statistical significance ($p<0.05$) determined by Student's t-test. Proteins with an H/L ratio greater than 1.5 are highlighted in red; those with a ratio greater than 1.3 are in pink and those with a ratio greater than 1.2 are in light pink. Proteins with an H/L ratio less than 0.7 are highlighted in green and those with an H/L ratio of 0.7 - 0.8 are highlighted in light green.
