# Supplementary material for: Overexpression of the double homeodomain protein DUX4c interferes with myofibrillogenesis and induces clustering of myonuclei
Source: Skelet Muscle. 2018 Jan 12;8:2. doi: 10.1186/s13395-017-0148-4 (PMC5767009; doi:10.1186/s13395-017-0148-4)
Supplement: Supplementary file 11 — DUX4 myogenic enhancer (DME1 and 2) might interact with the DUX4c gene. The DUX4 forward primer used in a 3C capture experiment [79] is highlighted in a common region in DUX4 and DUX4c ORF sharing 100% identity (Accession numbers AF AF117653 and AY500824). In Himeda et al. [79], RT-qPCR was performed using this primer in combination with either DIR1 (containing DME1) or DIR2 (containing DME2) primer to confirm DUX4-DME1 or DUX4-DME2 interactions. The BlgII site (boxed) used to digest chromatin-linked DNA regions is also shown and is located 226-bp downstream of the 5’end of the DUX4 primer. The region between DUX4 primer and the BglII site share 100% identity. (PDF 287 kb) [file 13395_2017_148_MOESM11_ESM.pdf]

Figure S10

```
DUX4   2599 GCCCCCTGGCTGCACCTGCCGCAGTGCACAGTCCGGCTGAGGTGCACGGG 2648
              |.|||||||||||||||||||||||||||.||||||||||||||||||
DUX4c   810 --CTCCTGGCTGCACCTGCCGCAGTGCACAGGCCGGCTGAGGTGCACGGG  857

DUX4   2649 AGCCCGCCGGCCTCTCTCTGCCCGCGTCCGTCCGTGAAATTCCGGCCGGG 2698
            ||||||||||||||||||||||||||||||||||||||||||||||||||
DUX4c   858 AGCCCGCCGGCCTCTCTCTGCCCGCGTCCGTCCGTGAAATTCCGGCCGGG  907

DUX4   2699 GCTCACCGCGATGGCCCTCCCGACACCCTCGGACAGCACCCTCCCCGCGG 2748
            |||||||||||||||||||||||||||.||||||||||||||||||||||
DUX4c   908 GCTCACCGCGATGGCCCTCCCGACACCTTCGGACAGCACCCTCCCCGCGG  957

DUX4   2749 AAGCCCGGGGACGAGGACGGCGACGGAGACTCGTTTGGACCCCGAGCCAA 2798
            ||||||||||||||||||||||||||||||||||||||||||||||||||
DUX4c   958 AAGCCCGGGGACGAGGACGGCGACGGAGACTCGTTTGGACCCCGAGCCAA 1007

DUX4   2799 AGCGAGGCCCTGCGAGCCTGCTTTGAGCGGAACCCGTACCCGGGCATCGC 2848
            ||||||||||||||||||||||||||||||||||||||||||||||||||
DUX4c  1008 AGCGAGGCCCTGCGAGCCTGCTTTGAGCGGAACCCGTACCCGGGCATCGC 1057

DUX4   2849 CACCAGAGAACGGCTGGCCCAGGCCATCGGCATTCCGGAGCCCAGGGTCC 2898
            ||||||||||||||||||||||||||||||||||||||||||||||||||
DUX4c  1058 CACCAGAGAACGGCTGGCCCAGGCCATCGGCATTCCGGAGCCCAGGGTCC 1107

DUX4   2899 AGATTTGGTTTCAGAATGAGAGGTCACGCCAGCTGAGGCAGCACCGGCGG 2948
            ||||||||||||||||||||||||||||||||||||||||||||||||||
DUX4c  1108 AGATTTGGTTTCAGAATGAGAGGTCACGCCAGCTGAGGCAGCACCGGCGG 1157

DUX4   2949 GAATCTCGGCCCTGGCCCGGGAGACGCGGCCCGCCAGAAGGCCGGCGAAA 2998
            ||||||||||||||||||||||||||||||||||||||||||||||||||
DUX4c  1158 GAATCTCGGCCCTGGCCCGGGAGACGCGGCCCGCCAGAAGGCCGGCGAAA 1207

DUX4   2999 GCGGACCGCCGTCACCGGATCCCAGACCGCCCTGCTCCTCCGAGCCTTTG 3048
            ||||||||||||||||||||||||||||||||||||||||||||||||||
DUX4c  1208 GCGGACCGCCGTCACCGGATCCCAGACCGCCCTGCTCCTCCGAGCCTTTG 1257

DUX4   3049 AGAAGGATCGCTTTCCAGGCATCGCCGCCCGGGAGGAGCTGGCCAGAGAG 3098
            ||||||||||||||||||||||||||||||||||||||||||||||||||
DUX4c  1258 AGAAGGATCGCTTTCCAGGCATCGCCGCCCGGGAGGAGCTGGCCAGAGAG 1307
                                         BglII
DUX4   3099 ACGGGCCTCCCGGAGTCCAGGATTCAGATCTGGTTTCAGAATCGAAGGGC 3148
            ||||||||||||||||||||||||||||||||||||||||||||||||||
DUX4c  1308 ACGGGCCTCCCGGAGTCCAGGATTCAGATCTGGTTTCAGAATCGAAGGGC 1357

DUX4   3149 CAGGCACCCGGGACAGGGTGGCAGGGCGCCCGCGCAGGCAGGCGGCCTGT 3198
            ||||||||||||||||||||||||||||||||||||||||||||||||||
DUX4c  1358 CAGGCACCCGGGACAGGGTGGCAGGGCGCCCGCGCAGGCAGGCGGCCTGT 1407

DUX4   3199 GCAGCGCGGCCCCCGGCGGGGGTCACCCTGCTCCCTCGTGGGTCGCCTTC 3248
            ||||||||||||||||||||||||||||||||||||||||||||||||||
DUX4c  1408 GCAGCGCGGCCCCCGGCGGGGGTCACCCTGCTCCCTCGTGGGTCGCCTTC 1457

DUX4   3249 GCCCACACCGGCGCGTGGGGAACGGGGCTTCCCGCACCCCACGTGCCCTG 3298
            ||||||||||||||||||||||||||||||||||||||||||||||||||
DUX4c  1458 GCCCACACCGGCGCGTGGGGAACGGGGCTTCCCGCACCCCACGTGCCCTG 1507
```

```
DUX4   3299 CGCGCCTGGGGCTCTCCCACAGGGGGCTTTCGTGAGCCAGGCAGCGAGGG 3348
            ||||||||||||||||||||||||||||||||||||||||||||||||||
DUX4c  1508 CGCGCCTGGGGCTCTCCCACAGGGGGCTTTCGTGAGCCAGGCAGCGAGGG 1557

DUX4   3349 CCGCCCCCGCGCTGCAGCCCAGCCAGGCCGCGCCGGCAGAGGGGATCTCC 3398
            ||||||||||||||||||||||||||||||||||||||||||||||||||
DUX4c  1558 CCGCCCCCGCGCTGCAGCCCAGCCAGGCCGCGCCGGCAGAGGGGATCTCC 1607

DUX4   3399 CAACCTGCCCCGGCGCGCGGGGATTTGGCCTACGCCGCCCCGGCTCCTCC 3448
            ||||||||||||||||||||||||||.|||||||||||||||||||||||
DUX4c  1608 CAACCTGCCCCGGCGCGCGGGGATTTCGCCTACGCCGCCCCGGCTCCTCC 1657

DUX4   3449 GGACGGGGCGCTCTCCCACCCTCAGGCTCCTCGGTGGCCTCCGCACCCGG 3498
            ||||||||||||||||||||||||||||||||||||||||||||||||||
DUX4c  1658 GGACGGGGCGCTCTCCCACCCTCAGGCTCCTCGGTGGCCTCCGCACCCGG 1707

DUX4   3499 GCAAAAGCCGGGAGGACCGGGACCCGCAGCGCGACGGCCTGCCGGGCCCC 3548
            ||||||||||||||||||||||||||||||||||||||||||||||||||
DUX4c  1708 GCAAAAGCCGGGAGGACCGGGACCCGCAGCGCGACGGCCTGCCGGGCCCC 1757

DUX4   3549 TGCGCGGTGGCACAGCCTGGGCCCGCTCAAGCGGGGCCGCAGGGCCAAGG 3598
            ||||||||||||||||||||||||||||||||||||||||||||||||||
DUX4c  1758 TGCGCGGTGGCACAGCCTGGGCCCGCTCAAGCGGGGCCGCAGGGCCAAGG 1807

DUX4   3599 GGTGCTTGCGCCACCCACGTCCCAGGGGAGTCCGTGGTGGGGCTGGGGCC 3648
            ||||||||||||||||||||||||||||||||||||||||||||||||||
DUX4c  1808 GGTGCTTGCGCCACCCACGTCCCAGGGGAGTCCGTGGTGGGGCTGGGGCC 1857

DUX4   3649 GGGGTCCCCAGGTCGCCGGGGCGGCGTGGGAACCCCAAGCCGGGGCAGCT 3698
            ||||||||||||||||||||||||||||||||||||||||||||||||||
DUX4c  1858 GGGGTCCCCAGGTCGCCGGGGCGGCGTGGGAACCCCAAGCCGGGGCAGCT 1907

DUX4   3699 CCACCTCCCCAGCCCGCGCCCCCGGACGCCTCCGCCTCCGCGCGGCAGGG 3748
            |||||||||||||||||||||||||||||||||||        ||||.|.
DUX4c  1908 CCACCTCCCCAGCCCGCGCCCCCGGACGCCTCCGC--------GGCAAGC 1949

DUX4   3749 GCAGATGCAAGGCATCCCGGCGCCCTCCCAGGCGCTCCAGGAGCCGGCGC 3798
            .|||||||.||.|||||.|||||| |||||..|||||||||||||||.||
DUX4c  1950 ACAGATGCCAGCCATCCAGGCGCC-TCCCAACCGCTCCAGGAGCCGGGGC 1998

DUX4   3799 CCTGGTCTGCACTCCCCTGCGGCCTGCTGCTGGATGAGCTCCTGGCGAGC 3848
            .||.||||.||.||.|||.|.|||||.|.   .|||||||||||..||..
DUX4c  1999 GCTCGTCTACAGTCACCTCCAGCCTGTTA---TATGAGCTCCTGTAGACA 2045

DUX4   3849 CCGGAGTTTCTGCAGCAGGCGCAACCTCTCCTAGAAACGGAGGCCCCGGG 3898
            ||.|||||||.|||..||||.|.||||.|||||||..|||.|.|.|.|||
DUX4c  2046 CCAGAGTTTCAGCAAAAGGCACGACCTTTCCTAGATCCGGCGCCACTGGG 2095

DUX4   3899 GGAGCTGGAGGCCTCGGAAGAGGCCGCCTCGCTGGAAGCACCCCTCAGCG 3948
            |||||||.|||.|..|||||||.||||...|||||||.|||.|||||||.
DUX4c  2096 GGAGCTGAAGGACGTGGAAGAGCCCGCTCTGCTGGAACCACTCCTCAGCC 2145

DUX4   3949 AGGAAGAATACCGGGCTCTGCTGGAGGAGCTTTAGGACGCGGGGTTGGGA 3998
            ||||||||.|||||||||||||||||||||.....|..|||||||||||.
DUX4c  2146 AGGAAGAACACCGGGCTCTGCTGGAGGAGCAGGTTGGAGCGGGGTTGGGG 2195
```
